# Ciliary Genes Are Down-Regulated in Bronchial Tissue of Primary Ciliary Dyskinesia Patients

**DOI:** 10.1371/journal.pone.0088216

**Published:** 2014-02-06

**Authors:** Maciej Geremek, Ewa Ziętkiewicz, Marcel Bruinenberg, Lude Franke, Andrzej Pogorzelski, Cisca Wijmenga, Michał Witt

**Affiliations:** 1 International Institute of Molecular and Cell Biology, Warsaw, Poland; 2 Genetics Department, University Medical Centre Groningen and University of Groningen, Groningen, the Netherlands; 3 Complex Genetics Group, Department of Biomedical Genetics, University Medical Center Utrecht, Utrecht, the Netherlands; 4 Department of Molecular and Clinical Genetics, Institute of Human Genetics, Poznan, Poland; 5 Institute of Tuberculosis and Lung Diseases, Rabka, Poland; Institute of Molecular and Cell Biology, Singapore

## Abstract

Primary ciliary dyskinesia (PCD) is a rare, genetically heterogeneous disease characterized by recurrent respiratory tract infections, sinusitis, bronchiectasis and male infertility. The pulmonary phenotype in PCD is caused by the impaired motility of cilia in the respiratory epithelium, due to ultrastructural defects of these organelles. We hypothesized that defects of multi-protein ciliary complexes should be reflected by gene expression changes in the respiratory epithelium. We have previously found that large group of genes functionally related to cilia share highly correlated expression pattern in PCD bronchial tissue. Here we performed an explorative analysis of differential gene expression in the bronchial tissue from six PCD patients and nine non-PCD controls, using Illumina HumanRef-12 Whole Genome BeadChips. We observed 1323 genes with at least 2-fold difference in the mean expression level between the two groups (t-test p-value <0.05). Annotation analysis showed that the genes down-regulated in PCD biopsies (602) were significantly enriched for terms related to cilia, whereas the up-regulated genes (721) were significantly enriched for terms related to cell cycle and mitosis. We assembled a list of human genes predicted to encode ciliary proteins, components of outer dynein arms, inner dynein arms, radial spokes, and intraflagellar transport proteins. A significant down-regulation of the expression of genes from all the four groups was observed in PCD, compared to non-PCD biopsies. Our data suggest that a coordinated down-regulation of the ciliome genes plays an important role in the molecular pathomechanism of PCD.

## Introduction

Cilia are small cellular projectiles, extending from the cell surface, with which they share the cell membrane. The ciliary axoneme is built on a scaffold of nine peripheral microtubule doublets, associated with many multi-protein complexes, including outer and inner dynein arms (ODA and IDA), nexin links and radial spokes. Cilia act either as sensors (primary cilia) or propel fluid over the epithelia of various organs (motile cilia) [1]; their dysfunction is the underlying cause of many systemic diseases.

Primary ciliary dyskinesia (PCD) is a rare genetic disease characterized by recurrent respiratory tract infections, bronchiectasis and infertility. Pulmonary symptoms occur because of the lack of an efficient mucociliary clearance, caused by the kinetic dysfunction of motile cilia in the respiratory epithelium. Male infertility is caused by the dysmotility of flagella in spermatozoids. *Situs inversus*, a mirror reversal of body organ positioning, is present in approximately half of the PCD patients because of the immotility of primary cilia of the embryonic node [2], [3]. In rare cases, PCD symptoms are a part of syndromic diseases, e.g. X-linked retinitis pigmentosa [4], [5] and oral-facial-digital type I syndrome [6]. The diagnosis of PCD is based on the clinical criteria, the delayed saccharin test [7], and low levels of nasal nitric oxide [8]. These symptoms are supported by the imaging studies of the respiratory cilia. Tissue specimens obtained through bronchoscopy are evaluated in the light microscope for ciliary beating and with the electron microscope for defects of the ciliary ultrastructure. Among a wide spectrum of various ultrastructural ciliary lesions documented in PCD patients [9], the lack of ODA and/or IDA is the most frequently observed defect.

Genetically, PCD is a largely autosomal recessive trait with extensive heterogeneity. To date, nineteen genes have been found to be mutated in PCD: *DNAH5* [10], *DNAH11* [11], *DNAI1* [12], *DNAI2* [13], *RSPH9* [14], *RSPH4A* [14], *TXNDC3* [15], *KTU/PF13* [16], *LRRC50* [17], [18], *CCDC39* [19], *CCDC40* [20], *CCDC103* [21], *HEATR2* [22], *LRRC6* [23], *DNAL1* [24], *DNAAF3* [25], *HYDIN* [26], *CCDC114* [27],*CCDC164* [28], *CCDC65* [29], *c21orf59* [30], *SPAG1* [31], *ZMYND10* [32], *RSPH1* [33], *ARMC4* [34], and *DYX1C1/DNAAF4* [35]; additional genes are involved in syndromic forms of PCD: *RPGR* [4], [5] and *OFD1* [6]. Products of most of the PCD-related genes have conserved orthologs in *Chlamydomonas reinhardtii* and have a well-defined ultrastructural localization in the axoneme. However, the large complexity of the cilium, which is composed of hundreds of proteins, renders studying the ciliary function a difficult task. In effect, the genetic basis of PCD in about half of patients remains unknown.

Previously, in search for a better understanding of the molecular basis of functional defects of cilia in PCD, we have performed the whole-genome expression profiling in bronchial biopsies from six PCD patients [36]. Clustering analysis revealed a large group of genes with highly correlated expression pattern in PCD biopsies, but not in controls; based on the series of in silico analyses, we have indicated over 200 new genes potentially involved in the biology of human cilia. In the present study, we further explored the gene expression profiling data to characterize patterns of differential gene expression in bronchial tissue from PCD patients and non-PCD controls. We report that the significant proportion of genes that are down-regulated in PCD encode specific elements of the cilium, suggesting that a coordinated down-regulation of the ciliome genes plays an important role in the molecular pathomechanism of PCD.

## Materials and Methods

### Ethics statement

The Institutional Review Board (IRB) of the Medical University of Poznań approved use of bronchial biopsy specimens of patients and controls for genetic studies on PCD. Specimens were collected during routine hospital procedures. Written informed consent was obtained from all subjects.

### Bronchial biopsies and subjects

Material for this study, obtained from six unrelated PCD patients and nine unrelated control individuals, has been described in detail in Geremek et al. [36]. Briefly, clinical evaluation of the PCD patients was performed at the Institute of Tuberculosis and Lung Diseases in Rabka, Poland, by the experienced physician (AP). The primary bronchopulmonary symptoms in the patients were: sinusitis, nasal polyps, bronchiectasis, and recurrent infections of the upper respiratory tract. PCD status was also indicated by the positive results of routine diagnostic tests: the delayed saccharine test and lack of the ciliary motility in light microscopy imaging. In four patients (#1, 2, 3, 4), PCD diagnosis was confirmed by the low level of NO (25–188 ppb), measured in the nasal cavity (chemiluminescence analyzer, the threshold value of 200 ppb) [8] (Table S1). Three of these patients (#1, 3, 4) had ODA/IDA defect. One patient (#6) for whom no NO data were available had IDA defect and another one (#5) in whom NO level was in the normal range had ODA/IDA defect. Two of the patients (#5, 6) had situs inversus. The mutational status was known for one patient (patient #4), who was a compound heterozygote for two mutations in *DNAH5*. Non-PCD controls were individuals referred to the Institute of Tuberculosis and Lung Diseases in Rabka for various reasons unrelated to PCD; they presented no symptoms of acute pulmonary disease, no bronchoscopic signs of the disturbance of mucociliary transport, and had normal ciliary beating in the light microscopy.

Biopsies were obtained during a diagnostic bronchoscopy, using the same protocol in PCD and non-PCD individuals. Specimens were stored in RNAlater buffer and used for RNA isolation.

### Gene expression analysis

The quality and concentration of RNA were determined using the 2100 Bioanalyzer (Agilent, Amstelveen, The Netherlands) with the Agilent RNA 6000 Nano Kit. Anti-sense RNA was synthesized, amplified and purified using the Ambion Illumina TotalPrep Amplification Kit (Ambion, USA) according to the manufacturer's protocol. Complementary RNA was hybridized to Illumina HumanRef-12 Whole Genome BeadChips and scanned on the Illumina BeadArray Reader. Data was handled through the Illumina BeadStudio Gene Expression module v3.2. The expression data has been submitted to GEO under accession number GSE25186.

### Data-analysis

Initial steps of data preprocessing, quantile normalization, background subtraction and quality control were performed using the BeadStudio software. The data were exported to GeneSpring 9. Expression values with a threshold value below 5 were raised to 5, and the data were scaled by a base 2 logarithm.

Ensembl Biomart module was used to obtain Ensemble gene IDs of the differentially expressed genes that were subject to annotation enrichment analysis and the Ciliome database (www.ciliome.com) [37] search. Fold-change analysis for all the probes was performed using GeneSpring 9 software. Genes that had at least 2-fold expression change (with the t-test p-value <0.05) in PCD patients as compared to the controls were subjected to gene annotation enrichment analysis using DAVID (Database for Annotation, Visualization, and Integrated Discovery) [38]. DAVID applies a modified Fisher exact test, to establish if the proportion of genes falling into an annotation category differs for a particular group of genes and the background group of genes; the p-values were corrected for multiple testing using the FDR method. The resulting annotation terms were then clustered into functional groups.

PubMed was used as references for known ciliary genes encoding components of radial spokes and intraflagellar transport proteins. Outer and inner dynein arms genes were taken from Pazour et al [39]. The R package was used for statistical calculations.

The log2-transformed mean expression values for the probes representing protein components of the specific ultrastructural ciliary elements were compared in the patients and controls, and the Wilcoxon Mann-Whitney test was used to obtain p-values for individual comparisons. In case of genes represented by multiple probes, mean values were used for calculations. The unweighted Z-method of combining probabilities from independent tests was used to assess if the distribution of p-values deviated from normal [40]. The Z-transform test converts one-tailed p-values from each of the independent tests into standard normal deviates; the sum of these standard normal deviates, divided by the square root of the number of tests, has a standard normal distribution if the common null hypothesis is true.

## Results

### Whole genome analysis (fold change and gene enrichment)

We identified 1323 genes (1396 probes), which displayed at least 2-fold difference in the expression level in PCD compared to non-PCD bronchial tissue (t-test p-value <0.05); 721of these genes (755 probes) were over-expressed, whereas 602 genes (641 probes) showed a reduced expression in PCD (Table S2).

To investigate the biological meaning of these differentially expressed genes, we performed annotation enrichment analysis, separately for the up-regulated and down-regulated genes, using DAVID software [38]. Annotations of the genes with the reduced expression in PCD patients were significantly enriched for terms related to microtubules, motility, cilia, and dyneins (Table 1). In the group of up-regulated genes, the highest scoring cluster was related to cell cycle and mitosis (Table 2). To investigate how many of the down- and up-regulated genes were functionally linked to cilia, the two groups (genes from Table S2) were used to query the Ciliome database [37]. This search revealed that 19% of the down-regulated and 11% of the up-regulated genes were related to cilia. Under a theoretical assumption that approximately 10% of the human genes are related to cilia (the Ciliome database contains 2024 entries), the proportion of ciliary genes found in the down-regulated group was significantly higher than in the whole set of human genes (binominal p-value 1.69e-08), while the list of up-regulated genes was not significantly enriched for genes related to cilia (p-value 0.26).

**Table 1:** Gene annotation analysis of the genes down-regulated in PCD (fold change >2.0, and p<0.05).

Annotation Cluster 1			
Enrichment Score: 7.25			
Ontology term	Count#	P_Value	FDR*
cilium	23	3.6E-14	1.1E-11
axoneme	15	6.2E-14	9.9E-12
microtubule	30	9.6E-13	1.0E-10
microtubule cytoskeleton	42	1.9E-12	1.5E-10
dynein complex	13	2.6E-12	1.6E-10
cell projection part	26	3.2E-11	1.3E-9
dynein heavy chain, N-terminal region 2	9	5.7E-10	4.1E-7
microtubule motor activity	14	9.2E-9	5.2E-6
microtubule associated complex	15	3.3E-8	8.0E-7
ciliary or flagellar motility	7	2.3E-7	3.8E-4
cytoskeleton	59	2.9E-7	6.6E-6
PIRSF002308:dynein heavy chain, ciliary	6	5.6E-7	8.0E-5
cilium biogenesis/degradation	7	2.4E-6	1.2E-4
ATPase, AAA+ type, core	12	3.5E-4	6.1E-2
non-membrane-bounded organelle	75	3.9E-3	6.3E-2
cell projection organization	16	1.2E-2	9.5E-1
cell motility	10	2.1E-1	1.0E0

Annotation Cluster 2			
Enrichment Score: 2.92			
Ontology term	Count#	P_Value	FDR*
nucleotide phosphate-binding region:ATP	39	4.6E-4	1.1E-1
purine nucleotide binding	63	6.1E-4	1.1E-1
ribonucleotide binding	60	9.9E-4	1.1E-1
nucleotide binding	69	1.9E-3	1.1E-1
adenyl ribonucleotide binding	50	2.0E-3	1.0E-1

Annotation Cluster 3			
Enrichment Score: 2.82			
Ontology term	Count#	P_Value	FDR*
cilium morphogenesis	7	5.9E-5	2.5E-2
cilium assembly	6	2.7E-4	8.9E-2
cell projection organization	16	1.2E-2	9.5E-1
cell part morphogenesis	12	2.1E-2	9.4E-1
cell projection assembly	6	3.2E-2	9.8E-1

# Count is a non-exclusive number of genes in each annotation category.

* False discovery rate.

**Table 2:** Gene annotation analysis of the genes up-regulated in PCD (fold change >2 and p<0.05).

Annotation Cluster 1			
Enrichment Score: 8.84			
Ontology term	Count#	P_Value	FDR*
cell cycle	47	7.6E-12	1.4E-8
M phase	30	8.5E-12	7.7E-9
mitotic cell cycle	29	7.0E-10	2.5E-7
cell division	22	6.0E-9	1.0E-6
nuclear division	21	1.0E-8	3.0E-6
organelle fission	21	2.0E-8	4.5E-6

Annotation Cluster 2			
Enrichment Score: 5.81			
Ontology term	Count#	P_Value	FDR*
intracellular non-membrane-bounded organelle	84	3.2E-8	9.6E-6
cytoskeleton	40	3.6E-3	5.3E-2

Annotation Cluster 3			
Enrichment Score: 4.93			
Ontology term	Count#	P_Value	FDR*
translational elongation	14	7.2E-8	1.3E-5
structural constituent of ribosome	16	4.5E-7	2.1E-4
large ribosomal subunit	10	3.2E-6	4.9E-4
ribonucleoprotein	18	6.8E-6	4.7E-4
translation	20	2.5E-5	3.0E-3
ribonucleoprotein complex	23	1.9E-4	5.6E-3

Annotation Cluster 4			
Enrichment Score: 3.86			
Ontology term	Count#	P_Value	FDR*
regulation of cell cycle	25	2.8E-8	5.7E-6
regulation of organelle organization	14	3.2E-4	3.1E-2
regulation of mitosis	6	4.5E-3	2.2E-1

Annotation Cluster 5			
Enrichment Score: 3.04			
Ontology term	Count#	P_Value	FDR*
condensed chromosome	12	2.3E-5	1.0E-3
chromosome	23	3.6E-5	1.3E-3
kinetochore	7	2.8E-3	4.5E-2
centromere	4	2.7E-2	3.9E-1

# Count is non-exclusive number of genes in each annotation category.

* False discovery rate.

### Known ciliary genes

To search for the evidence of differential expression of the genes related to specific elements of the axonemal ultrastructure, we assembled a list of human proteins represented on the microarray and predicted to be a part of the known ultrastructural ciliary components (ODA, IDA, radial spokes, intraflagellar transport-related proteins). We used the data from Pazour et al. [39], who applied phylogenetic analysis to assign human heavy dynein chains to ultrastructural components of outer and dyneins arms of flagella, and from Hom et al. [41], who compiled data on the nomenclature of dyneins. The assignment of the remaining genes was based on the PubMed search.

The resulting list of 37 human genes encoding proteins predicted to be a part of known ciliary components included: 8 ODA genes, 10 IDA genes, 8 radial spoke genes and 11 intraflagellar transport genes (Table 3). The log 2-transformed mean expression levels in PCD and non-PCD bronchial tissues, the fold change, the log2 of the fold change and the corresponding Wilcoxon Mann-Whitney test p-values are presented in Table 3. All the genes had a lower expression in PCD than in controls. Statistically significant evidence of the down-regulation in PCD was also observed when the combined p-values were calculated, using an unweighted Z-method [33], in four subgroups of the genes encoding ciliary components: ODA (p = 3.5e-08), IDA (p = 3.19e-12), radial spokes (p = 1.06e-4) and intraflagellar transport proteins (p = 1.27e-07), respectively (Table 3).

**Table 3:** Differential expression of the genes encoding outer dynein arms, inner dynein arms, radial spokes and intraflagellar transport proteins in the group of PCD cases compared to the non-PCD controls.

Gene^#^	PCD Log2 of the expression	Non-PCD Log2 of the expression	Regulation	Log2 of the fold change nonPCD/PCD	Fold change nonPCD/PCD	Wilcoxon Mann-Whitney one tail p-value	Z-method combined p-value
**Inner dynein arms**							**3.188885e-12**
DNAH1	7.099971713	8.823093021	Down	1.72312131	3.301499231	0.002397602	
DNAH10^#^	2.845467386	2.930532774	Down	0.08506539	1.060735818	0.297612503	
DNAH12L	2.984792693	4.691691968	Down	1.70689927	3.264584241	0.005496876	
DNAH2	8.605120088	10.73227718	Down	2.12715709	4.368557834	0.001398601	
DNAH3	5.76933297	8.094913833	Down	2.32558086	5.012675559	0.005605302	
DNAH7	6.514024915	8.269907386	Down	1.75588247	3.377328402	0.003796204	
DNHD2	8.860690672	10.45193877	Down	1.59124809	3.01309903	0.017982018	
DNHL1^#^	2.704540406	3.102569317	Down	0.39802891	1.317706357	0.069468652	
WDR63	6.410269672	8.011632917	Down	1.60136324	3.034298967	0.017982018	
WDR78^#^	4.278425094	5.226285631	Down	0.94786054	1.92900989	0.024775225	
**Outer dynein arms**							**3.524812e-08**
DNAH11	3.002922837	5.663426945	Down	2.66050411	6.322539346	0.002598476	
DNAH5	2.840433314	5.349519237	Down	2.50908592	5.692592858	0.010311612	
DNAH9^#^	4.195042679	4.979603695	Down	0.78456102	1.722568101	0.008791209	
DNAI1	8.674843985	9.844952993	Down	1.17010901	2.250286995	0.033166833	
DNAI2	7.177192762	8.6898654	Down	1.51267264	2.853381481	0.002397602	
DNAL1^#^	5.239162724	5.900496355	Down	0.66133363	1.58154393	0.163836164	
TCTE1	3.213232157	4.82357751	Down	1.61034535	3.053249212	0.007662144	
TCTE3	2.361725905	2.586822518	Down	0.22509661	1.168855518	0.617456403	
**Radial spokes**							**0.0001061203**
RSPH3	7.413315798	7.836831773	Down	0.42351598	1.341192183	0.227972028	
RSHL3 (RSPH4A)^#^	9.513876417	10.3676621	Down	0.85378569	1.807236971	0.136063936	
NME5 (RSPH23)	9.938877607	10.88896095	Down	0.95008335	1.931984273	0.264335664	
RSPH1	10.4069913	11.50297723	Down	1.09598593	2.137591135	0.072327672	
C6ORF206 (RSPH9)	8.57029857	9.296022759	Down	0.72572419	1.653730552	0.163836164	
RSPH10B^#^	4.839311246	5.755622796	Down	0.91631155	1.887284017	0.005994006	
PPIL6 (RSPH12)	6.766706564	8.011535825	Down	1.24482926	2.369905052	0.111888112	
DNAJB13	4.103136217	5.749770835	Down	1.64663462	3.131024115	0.033166833	
**Intraflagellar transport complex**							**1.275819e-07**
DYNC2H1	6.048332879	8.057745569	Down	2.00941269	4.026182839	0.002397602	
DYNC2LI1^#^	5.614105936	5.886374416	Down	0.27226848	1.20770532	0.264335664	
IFT122^#^	4.919297327	5.396632088	Down	0.47733476	1.39216939	0.008791209	
IFT140	6.501863507	7.674088316	Down	1.17222481	2.253589598	0.017982018	
IFT172	8.232366019	9.177805846	Down	0.94543983	1.925775902	0.017982018	
IFT52^#^	6.3875103	6.531748043	Down	0.14423774	1.105146586	0.303496503	
IFT57	8.12299147	9.303622084	Down	1.18063061	2.266758367	0.033166833	
IFT74	7.712753509	8.095831669	Down	0.38307816	1.304121386	0.227972028	
IFT80^#^	2.430337143	2.764181897	Down	0.33384475	1.260367755	0.100406593	
IFT81^#^	3.80548032	4.32006191	Down	0.51458159	1.428579763	0.194205794	
IFT88^#^	4.97817553	5.909540297	Down	0.93136477	1.907079214	0.033166833	

# In case of genes represented by multiple probes, mean values were used for calculations

The results were not dependent on individual PCD patients. Removing of each of the PCD subject did not significantly change the result of gene annotation analysis. Evidence of the down-regulation in four subgroups of the genes encoding ciliary components remained also significant.

## Discussion

A combination of the fold change with the significance measured by an uncorrected t-test (which takes into consideration the variance between the samples), has been shown to be a reliable and reproducible measure of differential expression [42].The gene expression profiling using the whole-genome Illumina panel, performed in bronchial biopsies from six PCD patients and nine non-PCD controls, revealed 1396 probes (representing 1323 genes) with at least 2 fold expression level difference between the PCD and non-PCD groups (t-test p-value <0.05). Functional annotation of the differentially expressed genes revealed that those down-regulated in PCD were related to microtubules, motility and cilia, and the up-regulated ones to the cell cycle and mitosis, i.e. to the processes that are related to microtubules by mitotic spindle and centrioles.

To further characterize the expression changes in PCD, we analyzed expression levels of a number of cilia-related genes represented on the chip. We assembled the list of 37 human genes representing: three subgroups, related to the ultrastructural components of motile cilia (outer dynein arms, inner dynein arms, and radial spokes), plus one functional subgroup (intraflagellar transport proteins). In all these subgroups, the reduced expression in bronchial biopsies from PCD patients was observed. The largest number of probes exhibiting significantly decreased expression was observed in the group of genes encoding components of inner and outer dynein arms.

Of the twenty-six genes known to be mutated in PCD, *CCDC39*, *CCDC164* and *SPAG1* were not represented on the array and *TXNDC3* was not stably expressed. Of the remaining twenty-two stably expressed genes (Table 4), ten were significantly down-regulated (>2-fold change and p<0.05). The majority of the down-regulated PCD genes encoded components of cilia ultrastructure: ODA (*DNAH11*, *DNAH5*, *DNAI1*, *DNAI2, DNAL1, CCDC114*); central pair appendix (*HYDIN*). The remaining down-regulated PCD genes encoded cytoplasmic proteins required for the assembling of dynein arms (*LRCC50* alias *DNAAF1, ZMYND10*). In twelve of the PCD genes the expression level did not significantly differ between PCD and non-PCD biopsies; five of these genes (*KTU* alias *DNAAF2, CCDC103*, *DYX1C1, c21orf59* and *C19ORF52* alias *DNNAAF3*) encoded cytoplasmic assembly proteins, two (*CCDC40* and *CCDC65*) encoded N-DRC (nexin-denein regulatory complex) proteins, one (*HEATR2*) encoded protein of an unknown location and three encoded radial spokes proteins (*RSPH4A, RSPH1* and *RSPH9*), one (*ARMC4*) encoded ODA docking component; only the expression of *DNAAF3* and *CCDC40* was slightly higher in PCD than in healthy controls. *ODF1* associated with the syndromic form of PCD, was expressed at the significantly lower lever in PCD patients. *DYX1C1, LRCC6, RPGR, RSPH1* had the fold change >2 but the p-values were between 0.05 and 0.1.

**Table 4:** Differential expression of the genes mutated in PCD and two genes mutated in syndromic disorders associated with PCD symptoms (*RPGR* and *ODF1*).

Gene#	Probe ID	PCD Log2 of the expression	Non-PCD Log2 of the expression	Regulation	Log2 of the fold change nonPCD/PCD	Fold change nonPCD/PCD	T-test p-value
ARMC4	4200274	8,327165	9,390315	down	1,06315	2,089488	0,130237
C19ORF52 (DNAAF3)	6290253	7.118648	6.746589	up	−0.372059	0.77267895	0.132469
C21ORF59*	7040162	10,05058	10,54353	down	0,492947	1,407317	0,212658
C6ORF206 (RSPH9)	840433	8.570299	9.296023	down	0.72572	1.653725749	0.135019
CCDC103	6770463	7.065492	7.691135	down	0.62564	1.542895124	0.240855
CCDC114	7210482	6.548517	8.35885	down	1.81033	3.507225031	0.017073
CCDC40*	4150072	2.886536	2.4908	up	−0.395737	0.76010098	0.150259
CCDC65	2370228	7,4877	8,417493	down	0,929793	1,905002	0,151083
DNAH11*	7330360	3.002923	5.663427	down	2.6605	6.322521334	0.004405
DNAH5	2350554	2.840433	5.349519	down	2.50909	5.692608957	0.003368
DNAI1	2810300	8.674844	9.844953	down	1.17011	2.250288539	0.040018
DNAI2	5960685	7.177193	8.689865	down	1.51267	2.85337626	0.006119
DNAL1*	6100682	3.770269	5.508203	down	1.73793	3.335562329	0.007245
DYX1C1*	3140523	4,361447	5,485308	down	1,123861	2,179294	0,071072
HEATR2*	5290037	8.210407	8.386503	down	0.1761	1.129825525	0.494105
HYDIN*	7550360	6.950931	9.07216	down	2.12123	4.350647105	0.000905
KTU (DNAAF2)	2490408	6.58843	6.791816	down	0.20339	1.151400704	0.579292
LRRC50 (DNAAF1)	5550035	8.577057	10.22783	down	1.65077	3.140011842	0.010513
LRRC6	2350477	6.796389	7.90332	down	1.10693	2.153868244	0.08437
ODF1	3120427	2.669272	3.925799	down	1.25653	2.38920393	0.046691
RPGR*	2750386	2.726082	3.783812	down	1.05773	2.081653578	0.065558
RSHL3 (RSPH4A)*	2630519	9.918507	10.80253	down	0.88402	1.845510569	0.187161
RSPH1	6020451	10,40699	11,50298	down	1,095986	2,137591	0,105587
ZMYND10	4540066	8,938617	10,15335	down	1,214728	2,320971	0,012449

* In case of genes represented by multiple probes, the most significant probe is shown.

# *TXNDC3* was not stably expressed, *CCDC39, CCDC164* and *SPAG1* were not represented on the array.

Given the lack of linear relation between RNA and protein, direct conclusions about a possible correlation between the RNA level and ultrastructural ciliary defects cannot be made. Large multi-protein complexes of the dynein arms are pre-assembled in the cytoplasm, before their intraflagellar transport to the ciliary axonemes. Immunohistochemical staining of the ciliated epithelium from PCD patients with identified mutations has shown that a defect/absence of one of the ciliary proteins may impair the delivery or assembly of other, non-mutated members of the axonemal substructures [43]. This relationship has been shown e.g. for mutated DNAH5 or DNAI1 and DNAH9 [43], mutated KTU and components of ODA/IDA [16], mutated CCDC40 or CCDC39 and components of IDA [19], [20], mutated LRRC6 and components of ODA/IDA [23]. Our data suggest that this impairment of the ciliary ultrastructure assembly may in fact originate at the transcription level; we hypothesize that a regulatory system may exist, which – in the presence of a mutated ciliary gene – down-regulates the expression of at least part of the ciliary genome. Such a regulation would play an important role in the molecular pathomechanism of PCD. During ciliogenesis the expression of ciliary genes is regulated by common transcription factors. The FOXJ1 transcription factor and RFX (regulatory factor X) family of transcription factors have been shown to regulate the expression of many cilia related genes. We have found *FOXJ1* (FC = 2,086838) and *RFX2* (FC = 2,412601) among the down-regulated genes indicating that the putative regulatory event acts upstream of these two transcription factors[44].

In our study, the strongest evidence of a significant down-regulation of the cilia-related genes in PCD bronchial biopsies concerned the genes predicted to encode elements of the inner and outer dynein arms and to a lesser extent, elements of radial spokes and proteins involved in ODA assembly or in the intraflagellar transport. The small study group provides only the proof of the concept and further studies are warranted. For example, to estimate the specificity of a putative regulatory system it will be important to repeat similar analyses using groups of patients carrying mutations in the same gene. In our small group of patients, only one of the patients had a known mutational status (two complementary heterozygous mutations in *DNAH5*). *DNAH5* is the strongest contributor to the genetic background of PCD, but one cannot exclude the possibility that there were mutations in other genes among the patients we examined. One may envision that the homogeneous group of patients with the mutations in the specific genes would cause different groups of the ciliary genes to undergo down-regulation.

Apart from the contribution to the understanding of the mechanisms of ciliary assembly, such studies may contribute to finding further candidate genes in PCD. To date, a number of candidate disease genes in several genomic regions reported to be linked to PCD remain to be identified; the failure of many linkage studies may be ascribed to the noise in linkage data, and difficulties in the functional prioritization of the candidate genes. In this context, observation of a down-regulation of the particular gene's expression in PCD might provide clues for selecting candidate genes from the linkage peaks.

## Supporting Information

Table S1 **Electron microscopy, NO measurements and situs status in the PCD patients studied.**
(DOCX)

Table S2 **Differentially expressed genes (fold change >2 and p<0.05).**
(DOC)
