# Supplementary material for: Ciliary Genes Are Down-Regulated in Bronchial Tissue of Primary Ciliary Dyskinesia Patients
Source: PLoS One. 2014 Feb 6;9(2):e88216. doi: 10.1371/journal.pone.0088216 (PMC3916409; doi:10.1371/journal.pone.0088216)
Supplement: Table S1 — Electron microscopy, NO measurements and situs status in the PCD patients studied. (DOCX) [file pone.0088216.s001.docx]

**Table S1.** **Electron microscopy, NO measurements and situs status in the PCD patients studied**

| Patient # | EM | NO (ppb) | *Situs inversus* |
| --- | --- | --- | --- |
| 1 | ODA/IDA | 67.1/120.0/90.1/133/177 | – |
| 2 | ND | 188/481/137 | – |
| 3 | ODA/IDA | 25.5/33 | – |
| 4* | ODA/IDA | 75.6/59.9/116 | – |
| 5 | ODA/IDA | 626/669/687 | + |
| 6 | IDA | ND | + |

*Two *DNAH5*  mutations; EM electron microscopy; ODA/IDA: lack of both outer and inner dynein arms; IDA: isolated lack of inner dynein arms; NO: nitric oxide measurements; ppb: parts per billion; ND: not determined.
