# Supplementary material for: Ciliary Genes Are Down-Regulated in Bronchial Tissue of Primary Ciliary Dyskinesia Patients
Source: PLoS One. 2014 Feb 6;9(2):e88216. doi: 10.1371/journal.pone.0088216 (PMC3916409; doi:10.1371/journal.pone.0088216)
Supplement: Table S2 — Differentially expressed genes (fold change >2 and p<0.05). (DOC) [file pone.0088216.s002.doc]

**Table S2**. **Differentially expressed genes (> 2-fold change p<0.05)**

| **Gene** | **ProbeID** | **Fold change** | **Regulation** | **t-test** |  | **Gene** | **ProbeID** | **Fold change** | **Regulation** | **t-test** |
| --- | --- | --- | --- | --- | --- | --- | --- | --- | --- | --- |
| IRS2 | 6110736 | 2,000081 | down | 0,018844 |  | LOC127602 | 3360088 | 2,000322 | up | 0,007265 |
| MTTP | 3520746 | 2,003799 | down | 0,029583 |  | TRIM47 | 840528 | 2,001623 | up | 0,020759 |
| PKIB | 3610615 | 2,004197 | down | 0,035131 |  | HS.544029 | 4200671 | 2,002011 | up | 0,005021 |
| C17ORF69 | 1070368 | 2,004541 | down | 0,014151 |  | HS.578256 | 3310239 | 2,002031 | up | 0,011475 |
| LOC550631 | 2810477 | 2,00641 | down | 0,01481 |  | HS.319180 | 2690671 | 2,002703 | up | 0,042412 |
| BBS5 | 160743 | 2,006851 | down | 0,04707 |  | LOC646990 | 6520209 | 2,002945 | up | 0,035902 |
| FAM134B | 3180661 | 2,008031 | down | 0,031341 |  | HERPUD2 | 3370554 | 2,004175 | up | 0,048717 |
| GPC4 | 6330270 | 2,010525 | down | 0,027257 |  | BTG3 | 4920053 | 2,005881 | up | 0,006158 |
| LOC440978 | 5960048 | 2,011607 | down | 0,021756 |  | HS.249577 | 6290438 | 2,006753 | up | 0,030412 |
| C6ORF85 | 6560328 | 2,014779 | down | 0,004573 |  | HS.543144 | 7510458 | 2,007606 | up | 0,040403 |
| L3MBTL | 7610685 | 2,01876 | down | 0,03232 |  | C17ORF91 | 5290161 | 2,008507 | up | 0,020013 |
| UCP2 | 6580059 | 2,021062 | down | 0,012568 |  | HS.545033 | 3360440 | 2,008581 | up | 0,020084 |
| PIGO | 3310451 | 2,021684 | down | 0,01117 |  | RPS21 | 2690338 | 2,009715 | up | 0,000251 |
| LOC642393 | 2260402 | 2,023422 | down | 0,040321 |  | DCX | 4810414 | 2,011051 | up | 0,029384 |
| GPR172B | 7510452 | 2,023879 | down | 0,012278 |  | HS.372983 | 2140450 | 2,011441 | up | 0,042696 |
| SART3 | 4830717 | 2,02421 | down | 0,036676 |  | DBR1 | 5960561 | 2,011478 | up | 0,021385 |
| FSD1L | 770646 | 2,027076 | down | 0,029869 |  | HS.573320 | 3400653 | 2,011776 | up | 0,024475 |
| KIAA1467 | 1580333 | 2,029357 | down | 0,025653 |  | LOC650546 | 1820458 | 2,011902 | up | 0,031533 |
| SMAP2 | 3930390 | 2,030036 | down | 0,017014 |  | LMTK3 | 6980632 | 2,012025 | up | 0,027787 |
| B3GNT6 | 2260220 | 2,030401 | down | 0,038284 |  | HS.433278 | 6660632 | 2,012438 | up | 0,000964 |
| MAN1A2 | 5050373 | 2,031104 | down | 0,015749 |  | CKMT1B | 5570021 | 2,012641 | up | 0,004533 |
| OAZ3 | 2650280 | 2,031124 | down | 0,041789 |  | HS.303060 | 780113 | 2,012889 | up | 0,025663 |
| LRRC45 | 3420440 | 2,031984 | down | 0,032867 |  | LOC388621 | 3370296 | 2,013737 | up | 0,00577 |
| C10ORF81 | 4290307 | 2,03238 | down | 0,001314 |  | LOC652777 | 6980168 | 2,013881 | up | 0,01032 |
| C21ORF7 | 150315 | 2,034127 | down | 0,016126 |  | MND1 | 7550730 | 2,014361 | up | 0,007959 |
| HS.207105 | 5360195 | 2,036819 | down | 0,027584 |  | MAF | 3610440 | 2,014904 | up | 0,046512 |
| WWC1 | 1090575 | 2,039234 | down | 0,002099 |  | FLJ43950 | 1500646 | 2,01505 | up | 0,008529 |
| HS.561786 | 870273 | 2,039333 | down | 0,021204 |  | GAGE12G | 1050056 | 2,015841 | up | 0,026356 |
| NPHP4 | 6280019 | 2,041768 | down | 0,033785 |  | LRRN3 | 2230538 | 2,016575 | up | 0,038137 |
| CYB5D1 | 6420040 | 2,041776 | down | 0,049121 |  | LOC648210 | 4760243 | 2,01736 | up | 0,002988 |
| LOC344595 | 2750593 | 2,042566 | down | 0,041092 |  | HS.583779 | 4640601 | 2,018029 | up | 0,011201 |
| ZBTB20 | 3440189 | 2,043338 | down | 0,008952 |  | CASP6 | 5550709 | 2,018204 | up | 0,028296 |
| CTF1 | 1190025 | 2,045392 | down | 0,020517 |  | LOC648732 | 2230458 | 2,018871 | up | 0,008763 |
| IQCA | 5050561 | 2,047014 | down | 0,03375 |  | DENND3 | 2320722 | 2,018892 | up | 0,047337 |
| DYNC2LI1 | 6520543 | 2,047847 | down | 0,046147 |  | ACBD5 | 4060435 | 2,018935 | up | 0,021614 |
| MAT1A | 4050112 | 2,049126 | down | 0,002572 |  | HS.563786 | 4730441 | 2,019495 | up | 0,0417 |
| STX5 | 6130364 | 2,049853 | down | 0,047917 |  | ACTR3 | 6940176 | 2,019562 | up | 0,031957 |
| HS.193784 | 3290609 | 2,052626 | down | 0,04528 |  | LOC642338 | 2640020 | 2,020514 | up | 0,00426 |
| FZD4 | 620255 | 2,052972 | down | 0,012294 |  | RGS10 | 3830092 | 2,020693 | up | 0,011933 |
| SV2B | 4540102 | 2,053165 | down | 0,010017 |  | LOC642362 | 1430440 | 2,021008 | up | 0,014766 |
| NAT14 | 60537 | 2,056108 | down | 0,00707 |  | LOC643684 | 1990332 | 2,021489 | up | 0,036875 |
| METT10D | 4670093 | 2,056879 | down | 0,042262 |  | LOC654230 | 2070671 | 2,023346 | up | 0,046964 |
| WNK1 | 5550682 | 2,060186 | down | 0,000234 |  | HS.132368 | 2070452 | 2,024653 | up | 0,046494 |
| ESRRG | 5080136 | 2,060814 | down | 0,019413 |  | LOC730262 | 1570022 | 2,024769 | up | 0,028954 |
| KIAA1622 | 7610411 | 2,061066 | down | 0,008378 |  | HEBP2 | 3940026 | 2,02501 | up | 3,86E-05 |
| PKD2 | 70161 | 2,061213 | down | 0,036761 |  | HS.575502 | 1410717 | 2,026185 | up | 0,00265 |
| MGLL | 3830327 | 2,062444 | down | 0,001189 |  | SNTA1 | 5690431 | 2,026715 | up | 0,020695 |
| LOC642559 | 5820538 | 2,063648 | down | 0,036309 |  | HS.545116 | 6110465 | 2,026723 | up | 0,04056 |
| GTF3C2 | 2640437 | 2,066207 | down | 0,000763 |  | HS.540902 | 7380133 | 2,026796 | up | 0,039254 |
| GP9 | 1050292 | 2,07085 | down | 0,045556 |  | LOC644266 | 3450142 | 2,028693 | up | 0,001532 |
| GNAL | 4200709 | 2,072844 | down | 0,04151 |  | LOC391352 | 2140435 | 2,02959 | up | 0,031022 |
| HDAC5 | 6620692 | 2,073983 | down | 0,047794 |  | HS.583646 | 1230128 | 2,029983 | up | 0,034389 |
| GARNL3 | 4880343 | 2,074229 | down | 0,022551 |  | HS.544493 | 2680341 | 2,030728 | up | 0,03056 |
| TNFAIP8L1 | 6200187 | 2,076249 | down | 0,037146 |  | EEF1B2 | 6330373 | 2,033783 | up | 0,009918 |
| GOLGB1 | 3130768 | 2,077199 | down | 0,025507 |  | PGK2 | 1400452 | 2,033824 | up | 0,002679 |
| LRRK1 | 510551 | 2,07784 | down | 0,032245 |  | PRB4 | 6560270 | 2,034023 | up | 0,047154 |
| FAM90A2P | 6960390 | 2,078937 | down | 0,005322 |  | HS.574968 | 6380653 | 2,034883 | up | 0,024961 |
| HS.575038 | 5900364 | 2,081429 | down | 0,049879 |  | SMCHD1 | 3390689 | 2,036024 | up | 0,017573 |
| FOXJ1 | 3170204 | 2,086838 | down | 0,032792 |  | PLAUR | 6220671 | 2,037295 | up | 0,008069 |
| KIAA1324 | 520609 | 2,087448 | down | 0,009025 |  | FLJ35785 | 2070634 | 2,037577 | up | 0,030281 |
| BZRAP1 | 6420646 | 2,088667 | down | 0,002055 |  | LOC137107 | 1780338 | 2,037659 | up | 0,042509 |
| AMPD3 | 150504 | 2,089158 | down | 0,001696 |  | SF3B14 | 3780544 | 2,037721 | up | 0,011176 |
| HEYL | 1070114 | 2,09049 | down | 0,03343 |  | LOC644403 | 5550154 | 2,038207 | up | 0,024297 |
| DIAPH2 | 5270181 | 2,091528 | down | 0,00474 |  | HS.539518 | 6660154 | 2,038723 | up | 0,023244 |
| PKN1 | 1660368 | 2,092619 | down | 0,040961 |  | LOC644362 | 2490246 | 2,040653 | up | 0,040113 |
| SYNE2 | 160121 | 2,092709 | down | 0,015029 |  | HS.176498 | 1660056 | 2,041444 | up | 0,035457 |
| ZNF468 | 650136 | 2,094985 | down | 0,030612 |  | HRIHFB2122 | 4880332 | 2,042195 | up | 0,046397 |
| GTF3C1 | 2750296 | 2,096368 | down | 0,00109 |  | LOC643807 | 60020 | 2,04257 | up | 0,049556 |
| HS.572968 | 2260735 | 2,096726 | down | 0,023214 |  | L1CAM | 1230735 | 2,043537 | up | 0,003033 |
| FAM104B | 6380148 | 2,096976 | down | 0,0053 |  | ZNRD1 | 4050673 | 2,046026 | up | 0,042116 |
| MTCH2 | 70689 | 2,09836 | down | 0,042074 |  | HS.65964 | 6550458 | 2,048048 | up | 0,038733 |
| HDAC10 | 6370520 | 2,099213 | down | 0,019308 |  | HS.333285 | 3460341 | 2,04874 | up | 0,036802 |
| SNORA7B | 7570110 | 2,102722 | down | 0,018109 |  | HS.569381 | 4010288 | 2,049139 | up | 0,002315 |
| CASZ1 | 4850184 | 2,105964 | down | 0,009616 |  | LOC152118 | 4920341 | 2,049482 | up | 0,029881 |
| MKS1 | 4260176 | 2,111133 | down | 0,007637 |  | LOC643855 | 6350746 | 2,049912 | up | 0,012926 |
| CDS1 | 5860010 | 2,111838 | down | 0,000479 |  | SPINK5L2 | 4780400 | 2,051154 | up | 0,045418 |
| ERO1LB | 2690672 | 2,116354 | down | 0,012276 |  | HS.543011 | 6250445 | 2,052288 | up | 0,004633 |
| FLJ45032 | 2120554 | 2,117384 | down | 0,030369 |  | DOCK2 | 10747 | 2,053206 | up | 0,045789 |
| SRGAP2 | 4860220 | 2,121745 | down | 0,033435 |  | HS.562230 | 780458 | 2,053497 | up | 0,026488 |
| FLJ21062 | 7160414 | 2,122585 | down | 0,009092 |  | LOC643997 | 2510022 | 2,053716 | up | 0,008142 |
| TTLL9 | 1780608 | 2,123296 | down | 0,038552 |  | FAM91A2 | 4830730 | 2,054043 | up | 0,032486 |
| MS4A8B | 580243 | 2,123546 | down | 0,014223 |  | LOC400682 | 6770433 | 2,054404 | up | 0,002461 |
| FLYWCH1 | 3390661 | 2,1245 | down | 0,026825 |  | LOC650278 | 2680445 | 2,054798 | up | 0,035913 |
| WDR19 | 6510397 | 2,124874 | down | 0,012424 |  | HS.548458 | 1070601 | 2,055453 | up | 0,016188 |
| LIG3 | 630047 | 2,127159 | down | 0,0142 |  | LOC644753 | 6100465 | 2,05661 | up | 0,005163 |
| LOC441155 | 2510100 | 2,130617 | down | 0,025391 |  | PDE3B | 5130471 | 2,057131 | up | 0,025009 |
| OSBPL3 | 6040181 | 2,133146 | down | 0,001504 |  | HS.544069 | 630239 | 2,057514 | up | 0,025797 |
| LOC440348 | 1230278 | 2,134586 | down | 0,022642 |  | BID | 1440037 | 2,057585 | up | 0,020966 |
| HS.23681 | 3120674 | 2,134741 | down | 0,020295 |  | HS.542579 | 840142 | 2,057681 | up | 0,0254 |
| BTBD16 | 5690102 | 2,135463 | down | 0,032841 |  | OSGEPL1 | 2060603 | 2,057964 | up | 0,020814 |
| MECP2 | 5130021 | 2,138038 | down | 0,024806 |  | C17ORF44 | 5810167 | 2,05816 | up | 0,038442 |
| MAP3K2 | 3800762 | 2,139739 | down | 0,000391 |  | C6ORF115 | 6020681 | 2,059089 | up | 0,010157 |
| ACCN2 | 1690050 | 2,143212 | down | 0,000628 |  | LOC643089 | 2750121 | 2,059577 | up | 0,044194 |
| MUC5AC | 1300240 | 2,14331 | down | 0,030669 |  | CXORF27 | 4180706 | 2,05996 | up | 0,001518 |
| KIF17 | 1090131 | 2,144147 | down | 0,014833 |  | CRLF1 | 4760095 | 2,060148 | up | 0,020167 |
| SLC46A3 | 2640324 | 2,145131 | down | 0,042807 |  | KIR3DL3 | 3780020 | 2,061064 | up | 0,031234 |
| EXPH5 | 7650286 | 2,145974 | down | 0,008487 |  | HS.30972 | 3370681 | 2,061149 | up | 9,96E-05 |
| HS.40061 | 4590379 | 2,147314 | down | 0,015467 |  | FLJ33544 | 4920315 | 2,061207 | up | 0,037702 |
| USP2 | 1850064 | 2,149957 | down | 0,044964 |  | FLJ13137 | 4850465 | 2,06314 | up | 0,034967 |
| CAPRIN1 | 2680730 | 2,150553 | down | 0,008049 |  | LIG4 | 2070093 | 2,064019 | up | 0,035539 |
| NAGA | 2070523 | 2,151025 | down | 0,019734 |  | HS.546059 | 2490243 | 2,065345 | up | 0,006019 |
| GSTM2 | 6550279 | 2,152518 | down | 0,033094 |  | CCDC64 | 5310753 | 2,065504 | up | 0,007365 |
| VLDLR | 5390661 | 2,155962 | down | 0,026132 |  | FXYD5 | 1470332 | 2,066901 | up | 0,02725 |
| 39878 | 780544 | 2,157364 | down | 0,032996 |  | ITGB2 | 3890373 | 2,067588 | up | 0,016248 |
| STEAP3 | 1300762 | 2,15767 | down | 0,000931 |  | SEC11B | 2600717 | 2,068272 | up | 0,011445 |
| MYO1E | 650379 | 2,158635 | down | 0,023582 |  | HS.541871 | 2030128 | 2,069999 | up | 0,035312 |
| ASL | 6620598 | 2,159516 | down | 0,029637 |  | HS.546213 | 6270072 | 2,070424 | up | 0,032768 |
| ULK2 | 460543 | 2,161386 | down | 0,021555 |  | LOC390483 | 7610435 | 2,071112 | up | 0,006545 |
| ZNF3 | 3290152 | 2,165807 | down | 0,048784 |  | IER3IP1 | 6860681 | 2,071209 | up | 0,007646 |
| HS.99203 | 5810601 | 2,166915 | down | 0,048341 |  | SUMO1P3 | 4050358 | 2,073189 | up | 0,010542 |
| HTF9C | 1300402 | 2,170575 | down | 0,025832 |  | KLF5 | 2230026 | 2,073388 | up | 0,03131 |
| KIAA1797 | 50544 | 2,172594 | down | 0,011652 |  | LOC340598 | 4150128 | 2,073498 | up | 0,02652 |
| TUBAL3 | 5670202 | 2,176543 | down | 0,033431 |  | LOC653073 | 610465 | 2,074494 | up | 0,045289 |
| RAGE | 1470348 | 2,177418 | down | 0,015633 |  | LOC649242 | 4900524 | 2,074804 | up | 0,004231 |
| CD99L2 | 1780079 | 2,178232 | down | 0,006334 |  | HS.544973 | 2970441 | 2,075871 | up | 0,029139 |
| PCDH7 | 4850373 | 2,183684 | down | 0,017489 |  | HS.202343 | 6130600 | 2,075964 | up | 0,007795 |
| PASK | 4150100 | 2,184397 | down | 0,007399 |  | LOC388621 | 730379 | 2,076042 | up | 0,004111 |
| LMTK2 | 1240270 | 2,1861 | down | 0,014145 |  | TCP11L1 | 1070753 | 2,076738 | up | 0,032465 |
| CAPRIN2 | 6200682 | 2,186341 | down | 0,008885 |  | NKX2-1 | 4060497 | 2,076805 | up | 0,035919 |
| AP4M1 | 2750168 | 2,187833 | down | 0,026714 |  | HS.542481 | 2350743 | 2,077013 | up | 0,021054 |
| HECW2 | 6290475 | 2,188559 | down | 0,00661 |  | ZNF16 | 730162 | 2,080768 | up | 0,048953 |
| C20ORF132 | 6980561 | 2,193406 | down | 0,023407 |  | CEP76 | 2600014 | 2,081367 | up | 0,010695 |
| SERHL | 5720379 | 2,194878 | down | 0,022166 |  | MAPK13 | 60349 | 2,083753 | up | 0,002853 |
| ZNF584 | 2480156 | 2,19531 | down | 0,009468 |  | BID | 1190022 | 2,084599 | up | 0,047826 |
| CC2D1B | 730497 | 2,195898 | down | 0,020381 |  | CDAN1 | 510386 | 2,084602 | up | 0,005177 |
| PAPPA | 730754 | 2,197229 | down | 0,040528 |  | SLC14A1 | 5560400 | 2,085057 | up | 0,009328 |
| TNFSF4 | 5690398 | 2,20113 | down | 0,015133 |  | RACGAP1 | 2190010 | 2,086247 | up | 0,037806 |
| RRBP1 | 1230307 | 2,201529 | down | 0,011338 |  | HS.146724 | 7400162 | 2,086278 | up | 0,021869 |
| C1ORF88 | 5420129 | 2,201773 | down | 0,041555 |  | HS.555388 | 4180593 | 2,086636 | up | 0,006716 |
| C9ORF45 | 1050050 | 2,203514 | down | 0,007168 |  | C2ORF32 | 2350142 | 2,088552 | up | 0,001628 |
| TTLL3 | 1260487 | 2,203544 | down | 0,003055 |  | FAHD1 | 650563 | 2,089938 | up | 0,022968 |
| PTPRM | 5860356 | 2,205416 | down | 0,030556 |  | SCARNA9 | 6220112 | 2,091637 | up | 0,034366 |
| HS.202577 | 240392 | 2,209405 | down | 0,031609 |  | C9ORF105 | 5310241 | 2,092497 | up | 0,033423 |
| C14ORF124 | 3310639 | 2,210523 | down | 0,047279 |  | MGC35361 | 1980370 | 2,09261 | up | 0,020942 |
| ZFP106 | 1010370 | 2,211026 | down | 0,014731 |  | UQCR | 4040747 | 2,092745 | up | 0,016147 |
| TMEM189-UBE2V1 | 4390220 | 2,211812 | down | 0,042342 |  | LOC653702 | 7610608 | 2,092823 | up | 0,011775 |
| ZNF574 | 580044 | 2,212146 | down | 0,026881 |  | PPIL5 | 10253 | 2,092915 | up | 0,008964 |
| SLC22A5 | 7050180 | 2,21234 | down | 0,000523 |  | LOC648099 | 5420349 | 2,094684 | up | 0,026158 |
| NLP | 5130703 | 2,213811 | down | 0,010334 |  | HS.161803 | 3190332 | 2,095849 | up | 0,022899 |
| CCDC13 | 5220301 | 2,215952 | down | 0,032268 |  | PPIL5 | 4830427 | 2,09642 | up | 0,015806 |
| C6ORF60 | 4490333 | 2,217022 | down | 0,019014 |  | MRPL1 | 2680435 | 2,096546 | up | 0,009742 |
| SIGIRR | 5860373 | 2,217346 | down | 0,010954 |  | MS4A6A | 2260129 | 2,096681 | up | 0,028933 |
| NFATC2IP | 4570369 | 2,217359 | down | 0,041068 |  | SCEL | 3460646 | 2,097129 | up | 0,025849 |
| DDAH1 | 3170292 | 2,217439 | down | 0,005985 |  | FCER1G | 3850440 | 2,098775 | up | 0,0083 |
| PDIA4 | 4060068 | 2,218635 | down | 0,009256 |  | ITGB2 | 7200156 | 2,101289 | up | 0,009883 |
| EFNB3 | 2680070 | 2,220331 | down | 0,019503 |  | C19ORF48 | 2850630 | 2,101702 | up | 0,025756 |
| RFX2 | 270136 | 2,224871 | down | 0,020818 |  | LOC643287 | 730142 | 2,102081 | up | 0,00296 |
| HS.4892 | 3420356 | 2,225971 | down | 0,008979 |  | KIAA0692 | 4560037 | 2,104292 | up | 0,005716 |
| C10ORF83 | 4060605 | 2,232574 | down | 0,001737 |  | TAX1BP3 | 1430647 | 2,106664 | up | 0,003234 |
| PPP1R10 | 6480474 | 2,235303 | down | 0,000788 |  | CAPG | 3840367 | 2,108809 | up | 0,046565 |
| CSPP1 | 3870608 | 2,236827 | down | 0,011873 |  | KIR2DL4 | 2140719 | 2,110351 | up | 0,008817 |
| PACS2 | 5550025 | 2,238855 | down | 0,025592 |  | LOC654164 | 2640136 | 2,111293 | up | 0,021752 |
| GLB1L | 2490360 | 2,243893 | down | 0,040428 |  | ACTC1 | 6940739 | 2,111576 | up | 0,014661 |
| NOMO1 | 2650040 | 2,244234 | down | 0,011978 |  | LOC642280 | 3710593 | 2,11219 | up | 0,015478 |
| MSH5 | 3130326 | 2,244374 | down | 0,017963 |  | YY1 | 6760017 | 2,112285 | up | 0,013729 |
| CUL7 | 2030541 | 2,248416 | down | 0,010364 |  | C1ORF51 | 2510068 | 2,112382 | up | 0,020815 |
| DNAI1 | 2810300 | 2,250287 | down | 0,040018 |  | LOC731640 | 2260168 | 2,11249 | up | 0,007538 |
| NSL1 | 5490112 | 2,251914 | down | 0,006854 |  | ERG | 6110601 | 2,114065 | up | 0,000309 |
| LOC653513 | 5820167 | 2,25285 | down | 0,005504 |  | HS.572268 | 1500615 | 2,114199 | up | 0,007761 |
| PGBD4 | 450400 | 2,252918 | down | 0,002384 |  | AP1S2 | 940706 | 2,114324 | up | 0,015778 |
| IFT140 | 6960376 | 2,25359 | down | 0,027001 |  | HS.156414 | 5090047 | 2,117646 | up | 0,029844 |
| C7ORF41 | 1740458 | 2,255037 | down | 0,002908 |  | C3ORF14 | 6560088 | 2,117886 | up | 0,00653 |
| NPAS2 | 6110167 | 2,259075 | down | 0,009101 |  | ANXA2P1 | 270609 | 2,117961 | up | 0,003318 |
| TDP1 | 3140181 | 2,260193 | down | 0,011075 |  | RFNG | 3370064 | 2,118085 | up | 0,042929 |
| NSUN7 | 2360563 | 2,260481 | down | 0,03506 |  | CCNA2 | 2650608 | 2,119302 | up | 0,018652 |
| NEK3 | 1190504 | 2,265365 | down | 0,03581 |  | HS.545094 | 2190292 | 2,119782 | up | 0,023557 |
| MUC4 | 110471 | 2,265668 | down | 0,02785 |  | HIST1H4C | 3890349 | 2,120496 | up | 0,005117 |
| COX19 | 1450255 | 2,265802 | down | 0,01696 |  | EDN1 | 7040386 | 2,12198 | up | 0,030899 |
| C10ORF95 | 1300735 | 2,265878 | down | 0,008162 |  | HS.158888 | 1570097 | 2,1227 | up | 0,02822 |
| IFT57 | 5700224 | 2,266758 | down | 0,038404 |  | HS.564510 | 10544 | 2,122846 | up | 0,00026 |
| LRRC23 | 10523 | 2,271962 | down | 0,042242 |  | HS.572260 | 4670128 | 2,122918 | up | 0,001716 |
| TEKT2 | 4570672 | 2,272609 | down | 0,01495 |  | HS.210390 | 1990064 | 2,123606 | up | 0,014208 |
| KIF24 | 2370546 | 2,274543 | down | 0,004477 |  | ZNF294 | 20398 | 2,125327 | up | 0,029537 |
| IGFBP5 | 2120524 | 2,274853 | down | 0,000608 |  | EDG8 | 1440750 | 2,125564 | up | 0,045517 |
| LIN52 | 10102 | 2,278306 | down | 0,001311 |  | LOC390367 | 4490156 | 2,126149 | up | 0,027367 |
| GLIS3 | 6560017 | 2,279218 | down | 0,004281 |  | HS.553971 | 6400400 | 2,127891 | up | 0,043501 |
| GCS1 | 4230309 | 2,280603 | down | 0,008896 |  | LOC647322 | 290093 | 2,1279 | up | 0,034088 |
| C9ORF117 | 6420372 | 2,283474 | down | 0,029741 |  | HS.560728 | 4880064 | 2,127912 | up | 0,037322 |
| TUBB2C | 2070368 | 2,286413 | down | 0,006866 |  | HS.583591 | 4490435 | 2,129733 | up | 0,021471 |
| FUS | 6130161 | 2,287638 | down | 0,03228 |  | LOC644952 | 2750162 | 2,132535 | up | 0,039504 |
| LOC283152 | 1850477 | 2,287859 | down | 0,048028 |  | GNA13 | 6650161 | 2,132829 | up | 0,039874 |
| XYLT1 | 630609 | 2,290545 | down | 0,049589 |  | C5ORF34 | 770102 | 2,133263 | up | 0,021204 |
| ARHGAP24 | 130008 | 2,290589 | down | 0,00579 |  | NKX2-1 | 940242 | 2,134902 | up | 0,047063 |
| HS.310040 | 7650689 | 2,290889 | down | 0,001181 |  | HS.584063 | 7050025 | 2,138013 | up | 0,004208 |
| SLC22A15 | 4480017 | 2,292801 | down | 0,030062 |  | SLC26A8 | 7610113 | 2,138256 | up | 0,007118 |
| PRICKLE2 | 1570168 | 2,294224 | down | 0,004117 |  | LOC554223 | 5560243 | 2,138681 | up | 0,010656 |
| ZFP106 | 3360619 | 2,29758 | down | 0,000776 |  | LOC728369 | 4730373 | 2,13979 | up | 0,001476 |
| HS.577265 | 2750605 | 2,298398 | down | 0,003411 |  | METRNL | 50095 | 2,14025 | up | 0,01621 |
| KIAA0256 | 2470762 | 2,302067 | down | 0,000495 |  | EEF1A2 | 6370356 | 2,140913 | up | 0,039635 |
| S100A13 | 5870577 | 2,302226 | down | 0,010821 |  | FHL2 | 6110025 | 2,142196 | up | 0,022067 |
| LOC654191 | 3990241 | 2,305831 | down | 0,020638 |  | HS.544625 | 160022 | 2,142201 | up | 0,049823 |
| HS.161875 | 1170348 | 2,306072 | down | 0,037568 |  | CNBP | 7380068 | 2,142332 | up | 0,006537 |
| NFAT5 | 3120136 | 2,306274 | down | 0,019417 |  | C20ORF79 | 5260142 | 2,145416 | up | 0,009629 |
| SAMD1 | 1440438 | 2,307578 | down | 0,019674 |  | MCART6 | 5820037 | 2,145929 | up | 0,043397 |
| DALRD3 | 650273 | 2,308433 | down | 0,027081 |  | LOC654121 | 1230554 | 2,146249 | up | 0,006108 |
| KLKB1 | 7400240 | 2,310588 | down | 0,049085 |  | HS.563525 | 2510544 | 2,147222 | up | 0,007911 |
| HIPK2 | 10332 | 2,314608 | down | 0,01403 |  | C10ORF116 | 6290168 | 2,150019 | up | 0,034195 |
| MUSTN1 | 3940368 | 2,314942 | down | 0,001953 |  | RPL32 | 2030681 | 2,150232 | up | 0,028603 |
| EPHX1 | 6900563 | 2,315119 | down | 0,002715 |  | C3ORF14 | 6650079 | 2,150858 | up | 0,00295 |
| SYT7 | 5050390 | 2,315337 | down | 0,016386 |  | LOC440731 | 4010270 | 2,152357 | up | 0,011322 |
| TTLL3 | 870500 | 2,315681 | down | 0,004188 |  | HS.542756 | 5960020 | 2,154931 | up | 0,037189 |
| KIAA1407 | 3140689 | 2,316578 | down | 0,020487 |  | LOC390354 | 510397 | 2,158533 | up | 0,000142 |
| ZMYND10 | 4540066 | 2,320971 | down | 0,012449 |  | HS.514877 | 1410328 | 2,15961 | up | 0,007875 |
| SLC23A1 | 5090279 | 2,324279 | down | 0,008683 |  | 40060 | 3420639 | 2,159914 | up | 0,027292 |
| HS.562534 | 6290632 | 2,32428 | down | 0,005165 |  | HS.540857 | 5360465 | 2,161137 | up | 0,034517 |
| SEMA3A | 6900114 | 2,326531 | down | 0,015253 |  | VPS24 | 2140634 | 2,161314 | up | 0,002769 |
| FILIP1 | 6200600 | 2,32879 | down | 0,025333 |  | LOC649075 | 4670440 | 2,161629 | up | 0,024275 |
| SOAT1 | 6200386 | 2,334738 | down | 0,030086 |  | TCL6 | 4670156 | 2,161634 | up | 0,0162 |
| ANKHD1-EIF4EBP3 | 520008 | 2,340195 | down | 0,001451 |  | OR13A1 | 1780465 | 2,162174 | up | 0,006038 |
| SMG6 | 7160563 | 2,342649 | down | 0,031164 |  | HS.567106 | 3800328 | 2,163309 | up | 0,036412 |
| FLJ36208 | 630553 | 2,345562 | down | 0,037153 |  | HS.242717 | 6220544 | 2,163964 | up | 0,018431 |
| DHRSX | 5270762 | 2,349124 | down | 0,007385 |  | LOC651029 | 7510593 | 2,166062 | up | 0,010545 |
| INHBB | 50446 | 2,350459 | down | 0,032096 |  | LOC442454 | 5890176 | 2,166881 | up | 0,021325 |
| CCDC69 | 160068 | 2,352493 | down | 0,022603 |  | HS.534809 | 1240458 | 2,167052 | up | 0,021313 |
| TOB2 | 1400491 | 2,355643 | down | 0,007887 |  | RPS28 | 650349 | 2,167135 | up | 7,35E-05 |
| NQO1 | 5360347 | 2,355762 | down | 0,031827 |  | EMP3 | 3800452 | 2,167499 | up | 0,005131 |
| AGPAT9 | 2060477 | 2,356382 | down | 0,024479 |  | HS.565597 | 1400014 | 2,173149 | up | 0,047342 |
| UCK1 | 2510070 | 2,358858 | down | 0,007652 |  | HS.335362 | 6220707 | 2,173438 | up | 0,010072 |
| LOC645863 | 4890072 | 2,359947 | down | 0,037402 |  | HS.134521 | 5080397 | 2,175771 | up | 0,011698 |
| VPS39 | 2710204 | 2,361734 | down | 0,002814 |  | LOC345537 | 4280537 | 2,179135 | up | 0,002218 |
| LOC650274 | 6760066 | 2,365392 | down | 0,035128 |  | TRIM7 | 6330397 | 2,181452 | up | 0,009855 |
| ATP9A | 6480630 | 2,367172 | down | 0,000307 |  | LOC388654 | 2190546 | 2,182667 | up | 0,00314 |
| DUSP18 | 3870408 | 2,367209 | down | 0,013733 |  | HS.553344 | 4540156 | 2,182961 | up | 0,039107 |
| MAOB | 4060433 | 2,369403 | down | 0,021428 |  | HS.544713 | 2450673 | 2,184849 | up | 0,003806 |
| ZNF626 | 5340068 | 2,369636 | down | 0,018236 |  | SNTG2 | 7570097 | 2,186314 | up | 0,007816 |
| AP2B1 | 2340451 | 2,373703 | down | 0,003183 |  | NME4 | 2320079 | 2,186684 | up | 0,001152 |
| HS.121623 | 430326 | 2,374742 | down | 0,026564 |  | TRUB2 | 5690121 | 2,188984 | up | 0,00852 |
| AHSA2 | 1170220 | 2,376503 | down | 0,005558 |  | CKMT1A | 3420661 | 2,190175 | up | 0,038388 |
| GAS7 | 1070435 | 2,377778 | down | 0,014512 |  | LOC283345 | 6840324 | 2,190176 | up | 0,002832 |
| HHAT | 2360639 | 2,385531 | down | 0,039277 |  | HS.380543 | 4900041 | 2,190476 | up | 0,021136 |
| CTSH | 4670162 | 2,387617 | down | 0,031143 |  | LOC649501 | 4900450 | 2,191957 | up | 0,040949 |
| ODF1 | 3120427 | 2,389199 | down | 0,046691 |  | HS.520420 | 7560441 | 2,193568 | up | 0,032705 |
| C1ORF104 | 3870091 | 2,39133 | down | 0,012135 |  | HS.544576 | 2070603 | 2,195028 | up | 0,002234 |
| FLJ90757 | 6130300 | 2,39331 | down | 0,032363 |  | ATP5G3 | 6220541 | 2,195188 | up | 0,006876 |
| MPDZ | 5270326 | 2,394402 | down | 0,024192 |  | VPS24 | 6200332 | 2,196065 | up | 0,012438 |
| USP34 | 3780138 | 2,39504 | down | 0,031755 |  | MGC61598 | 3800482 | 2,19623 | up | 0,040158 |
| DPY19L2 | 780386 | 2,401338 | down | 0,004054 |  | RPS15A | 7100717 | 2,196348 | up | 0,0265 |
| SDK1 | 10541 | 2,403044 | down | 0,023971 |  | PSMG1 | 2450136 | 2,196545 | up | 0,021108 |
| HKR1 | 6400753 | 2,407053 | down | 0,015894 |  | C5ORF20 | 2000162 | 2,19669 | up | 0,014779 |
| UFD1L | 730100 | 2,407638 | down | 0,003338 |  | HELLS | 7040161 | 2,197121 | up | 0,031617 |
| SEZ6L2 | 1090521 | 2,408476 | down | 0,008665 |  | HS.126101 | 4180671 | 2,197504 | up | 0,009273 |
| LOC654096 | 5050538 | 2,410537 | down | 0,003504 |  | MBIP | 5360411 | 2,197853 | up | 0,013025 |
| LOC730704 | 1990403 | 2,411477 | down | 0,049415 |  | CKS1B | 4210088 | 2,19881 | up | 0,001904 |
| RFX2 | 1470673 | 2,412601 | down | 0,007698 |  | LOC652703 | 1710440 | 2,199412 | up | 0,024746 |
| RNF41 | 1090433 | 2,41267 | down | 0,013257 |  | EIF3M | 940398 | 2,199482 | up | 0,002201 |
| SPAG1 | 6280296 | 2,418366 | down | 0,030276 |  | PMS2L2 | 1410064 | 2,20062 | up | 0,028139 |
| CLCN6 | 2900768 | 2,419179 | down | 0,021894 |  | HS.566310 | 6380253 | 2,202793 | up | 0,00999 |
| NSUN7 | 5870431 | 2,422659 | down | 0,025964 |  | GGH | 160615 | 2,20308 | up | 0,04545 |
| RBBP9 | 4730044 | 2,427691 | down | 0,005503 |  | PKIA | 510435 | 2,203277 | up | 0,023549 |
| ST5 | 6510307 | 2,428893 | down | 0,024772 |  | LOC401720 | 4760097 | 2,205695 | up | 0,009705 |
| HOOK3 | 3440241 | 2,430122 | down | 0,01258 |  | C5ORF29 | 6480079 | 2,205844 | up | 0,045682 |
| CES2 | 3850471 | 2,438729 | down | 0,028136 |  | OCIAD2 | 3310025 | 2,206041 | up | 0,005792 |
| LAMC2 | 4150725 | 2,441019 | down | 0,04421 |  | HS.584515 | 6770554 | 2,20718 | up | 0,031536 |
| IGFBP5 | 2190674 | 2,445042 | down | 0,001432 |  | COX7B | 2260576 | 2,207479 | up | 0,000625 |
| SCGB1A1 | 1450368 | 2,460368 | down | 0,028097 |  | HS.541868 | 4830743 | 2,208563 | up | 0,024142 |
| LOC654000 | 5700392 | 2,462979 | down | 0,003363 |  | LOC91561 | 2490450 | 2,20992 | up | 0,002298 |
| TTC21A | 580240 | 2,464344 | down | 0,005335 |  | PPCS | 2570725 | 2,210312 | up | 0,031315 |
| TTLL1 | 5290600 | 2,464613 | down | 0,009633 |  | PM20D2 | 50470 | 2,211447 | up | 0,03734 |
| C5AR1 | 6450092 | 2,468674 | down | 0,009621 |  | LOC554206 | 2570397 | 2,211617 | up | 0,042858 |
| SNCAIP | 1660019 | 2,47052 | down | 0,02549 |  | LOC652076 | 6840731 | 2,21171 | up | 0,049719 |
| KCNE1 | 2470554 | 2,474496 | down | 0,046501 |  | RPL34 | 2650524 | 2,211712 | up | 0,003061 |
| MAPRE3 | 6220026 | 2,475729 | down | 0,009416 |  | COMMD10 | 6510719 | 2,212396 | up | 0,04073 |
| SRGAP3 | 6350039 | 2,479085 | down | 0,002934 |  | C6ORF48 | 6280601 | 2,213597 | up | 0,000241 |
| C19ORF36 | 2000075 | 2,481331 | down | 0,001922 |  | HS.126651 | 2140731 | 2,214128 | up | 0,040312 |
| PTPRU | 3610609 | 2,481672 | down | 0,042515 |  | HS.579715 | 6060487 | 2,214164 | up | 0,00737 |
| HS.559604 | 2030180 | 2,484411 | down | 0,047364 |  | S100A4 | 3990458 | 2,216113 | up | 0,001195 |
| FAM81A | 7000494 | 2,484522 | down | 0,017927 |  | C8ORF59 | 1510452 | 2,216313 | up | 0,003319 |
| HS.147562 | 1450215 | 2,485051 | down | 0,025995 |  | HS.578016 | 270441 | 2,21858 | up | 0,040135 |
| HS.128847 | 7040612 | 2,486294 | down | 0,018291 |  | LOC642197 | 5130601 | 2,218837 | up | 0,001586 |
| KIAA0753 | 3830082 | 2,488568 | down | 5,22E-05 |  | TSPO | 4220091 | 2,219598 | up | 0,009042 |
| HHLA3 | 2230703 | 2,488913 | down | 0,015146 |  | TMEM11 | 360161 | 2,220331 | up | 0,04144 |
| CAPRIN2 | 1980202 | 2,492803 | down | 0,014651 |  | LOC652256 | 7040458 | 2,220932 | up | 0,011631 |
| C1ORF92 | 5820053 | 2,496239 | down | 0,020655 |  | NOP5/NOP58 | 2190189 | 2,22362 | up | 0,044728 |
| LRRC51 | 4230482 | 2,498376 | down | 0,041281 |  | LOC650465 | 3520739 | 2,226774 | up | 0,010238 |
| GALK2 | 4070706 | 2,499127 | down | 0,00153 |  | HS.513113 | 6650162 | 2,226774 | up | 0,042299 |
| CD97 | 1470626 | 2,502476 | down | 0,004951 |  | HS.552502 | 5910441 | 2,227331 | up | 0,003956 |
| GAS2L2 | 10193 | 2,508223 | down | 0,027943 |  | LOC651963 | 5220064 | 2,230121 | up | 0,030711 |
| SORD | 540246 | 2,514244 | down | 0,01665 |  | NMNAT3 | 1980129 | 2,231029 | up | 0,01513 |
| NEK11 | 3140168 | 2,514974 | down | 0,006583 |  | C11ORF17 | 3180288 | 2,231171 | up | 0,011389 |
| MC1R | 520292 | 2,515897 | down | 0,036586 |  | SPI1 | 5810398 | 2,234214 | up | 0,037777 |
| AKR1C4 | 2650736 | 2,516328 | down | 0,04171 |  | HS.440533 | 5560441 | 2,236814 | up | 0,043444 |
| FLJ42177 | 3420482 | 2,516416 | down | 0,026124 |  | HS.534529 | 3610008 | 2,236947 | up | 0,002746 |
| GATS | 2190541 | 2,52414 | down | 0,001633 |  | LOC728481 | 6900458 | 2,237372 | up | 0,000665 |
| FANK1 | 50296 | 2,528459 | down | 0,035339 |  | HSPE1 | 110110 | 2,237518 | up | 0,036229 |
| ODF2 | 1190066 | 2,529549 | down | 0,036358 |  | LOC654135 | 1070075 | 2,23759 | up | 0,015593 |
| C14ORF73 | 2060315 | 2,53057 | down | 0,037269 |  | POSTN | 510246 | 2,237843 | up | 0,03175 |
| RABL2B | 2940048 | 2,53074 | down | 0,026031 |  | PRR16 | 10324 | 2,239641 | up | 0,004248 |
| CCDC17 | 4830494 | 2,530794 | down | 0,04432 |  | EIF1AX | 3930296 | 2,240489 | up | 0,004244 |
| SLC16A5 | 650612 | 2,535292 | down | 0,000525 |  | PTTG3 | 5960224 | 2,241535 | up | 0,010126 |
| LOC441869 | 1980041 | 2,540311 | down | 0,01844 |  | FAM3B | 6270743 | 2,241699 | up | 0,007406 |
| C3ORF15 | 1010739 | 2,541131 | down | 0,028472 |  | HS.471011 | 730731 | 2,243352 | up | 0,038509 |
| TCF2 | 3520632 | 2,54756 | down | 0,046569 |  | LOC642960 | 1300577 | 2,244322 | up | 0,025225 |
| TMEM63B | 5130327 | 2,548537 | down | 0,046389 |  | RFC5 | 4890731 | 2,250175 | up | 0,035473 |
| FCN3 | 1170274 | 2,549117 | down | 0,030648 |  | OR2B3P | 5260762 | 2,250655 | up | 0,008207 |
| FRMPD2L1 | 7200187 | 2,549775 | down | 0,03023 |  | CTAGE6 | 5910170 | 2,252405 | up | 0,020922 |
| FLJ46082 | 6270246 | 2,550634 | down | 0,037204 |  | LOC731496 | 3310014 | 2,254937 | up | 0,016826 |
| MYH14 | 5490255 | 2,552424 | down | 0,0119 |  | SYT7 | 1710328 | 2,255439 | up | 0,01728 |
| AGPAT4 | 3440025 | 2,557587 | down | 0,040249 |  | HS.566481 | 520328 | 2,25548 | up | 0,034264 |
| RABL2B | 2360156 | 2,559406 | down | 0,001789 |  | SLC30A6 | 1340072 | 2,256596 | up | 0,020778 |
| MUC13 | 6940270 | 2,559466 | down | 0,03119 |  | RPL10A | 4040100 | 2,256681 | up | 0,018594 |
| C1ORF158 | 6100754 | 2,567931 | down | 0,025116 |  | HS.441649 | 460332 | 2,256887 | up | 0,005911 |
| RABL2A | 3390445 | 2,571456 | down | 0,018949 |  | HS.578028 | 780639 | 2,258312 | up | 0,041725 |
| ZNF3 | 3060215 | 2,571709 | down | 0,011774 |  | HS.434335 | 2070471 | 2,259164 | up | 0,019169 |
| STRA6 | 6420215 | 2,57227 | down | 0,01006 |  | NOL8 | 2120017 | 2,259633 | up | 0,028048 |
| HS.426929 | 3400273 | 2,57488 | down | 0,016963 |  | HS.121392 | 4560445 | 2,26076 | up | 0,003625 |
| HS.557356 | 4590187 | 2,577641 | down | 0,003468 |  | LOC653748 | 6660328 | 2,261397 | up | 0,015583 |
| HS.245791 | 430064 | 2,580247 | down | 0,022663 |  | TUBA4A | 4760474 | 2,262097 | up | 0,025944 |
| HEMK1 | 3420767 | 2,582174 | down | 0,00163 |  | ALOX5 | 1780273 | 2,262819 | up | 0,002131 |
| LRRC46 | 6960204 | 2,586737 | down | 0,008408 |  | LOC653899 | 2970328 | 2,263586 | up | 0,025496 |
| HYDIN | 1070307 | 2,588699 | down | 0,027814 |  | CD83 | 5050162 | 2,263674 | up | 0,034794 |
| MEGF10 | 270201 | 2,588944 | down | 0,027681 |  | LOC650298 | 670010 | 2,2638 | up | 0,032798 |
| CDK5RAP2 | 4260017 | 2,59014 | down | 0,017708 |  | SPINK5 | 6380707 | 2,264025 | up | 0,025551 |
| PTPRU | 6960577 | 2,592543 | down | 0,013375 |  | BTG3 | 7210605 | 2,264415 | up | 0,018604 |
| MFSD2 | 3840196 | 2,595184 | down | 0,019336 |  | LOC645737 | 4810408 | 2,264858 | up | 0,006922 |
| LOC644330 | 5810709 | 2,596222 | down | 0,028931 |  | LOC728012 | 4640593 | 2,265091 | up | 0,007666 |
| KLHL32 | 3440392 | 2,59646 | down | 0,021832 |  | NR2F2 | 6020458 | 2,265267 | up | 0,000829 |
| GRIN3B | 2640168 | 2,600014 | down | 0,040285 |  | SYNPO2 | 270743 | 2,266972 | up | 0,011031 |
| ZDHHC23 | 6590523 | 2,601907 | down | 0,006777 |  | TK1 | 4730196 | 2,267114 | up | 0,025891 |
| LOC286208 | 3180468 | 2,602661 | down | 0,005007 |  | HS.372922 | 2970435 | 2,267991 | up | 0,014813 |
| PCDP1 | 2810243 | 2,603153 | down | 0,028261 |  | PHF19 | 2570091 | 2,268292 | up | 0,034472 |
| CYP46A1 | 1770484 | 2,606753 | down | 0,000151 |  | CTSW | 6580408 | 2,269671 | up | 0,036664 |
| SLC41A1 | 580332 | 2,606775 | down | 0,000759 |  | ALG14 | 50128 | 2,270441 | up | 0,047711 |
| HYDIN | 4220435 | 2,610768 | down | 0,042513 |  | LOC643265 | 1260056 | 2,276356 | up | 0,01173 |
| SPATA18 | 2490215 | 2,611895 | down | 0,015525 |  | BOLA2 | 1740717 | 2,280315 | up | 0,017981 |
| FXYD1 | 5860022 | 2,612348 | down | 0,033302 |  | HS.177532 | 4560408 | 2,28047 | up | 0,001129 |
| ALDH3B1 | 2970356 | 2,613286 | down | 0,001406 |  | HS.545008 | 3800239 | 2,280664 | up | 0,02765 |
| HS.59203 | 3060392 | 2,619966 | down | 0,029592 |  | HS.287989 | 1230133 | 2,281077 | up | 0,011653 |
| INTU | 1300048 | 2,621674 | down | 0,021759 |  | LOC653874 | 7330301 | 2,284487 | up | 0,027359 |
| HS.400256 | 1980204 | 2,62293 | down | 0,000903 |  | DEPDC1 | 6200201 | 2,285246 | up | 0,025349 |
| C21ORF58 | 2070220 | 2,625375 | down | 0,037272 |  | LOC648605 | 2570400 | 2,286299 | up | 0,003819 |
| C6ORF185 | 3990603 | 2,62622 | down | 0,014101 |  | HS.538176 | 1850386 | 2,286305 | up | 0,025879 |
| PRKY | 1850687 | 2,634089 | down | 0,037121 |  | HS.564168 | 4880653 | 2,287364 | up | 0,043204 |
| TPPP | 5390541 | 2,637438 | down | 0,031992 |  | LOC388906 | 6480653 | 2,288753 | up | 0,001757 |
| STOML3 | 6380280 | 2,639429 | down | 0,048738 |  | FOXM1 | 990730 | 2,289367 | up | 0,046591 |
| LOC440836 | 6770132 | 2,644986 | down | 0,031992 |  | HS.572312 | 5490441 | 2,290353 | up | 0,012536 |
| CCDC78 | 6450487 | 2,646083 | down | 0,04102 |  | DFFB | 3830095 | 2,29093 | up | 0,032496 |
| SMYD2 | 1690347 | 2,64953 | down | 0,005748 |  | HS.572468 | 7400458 | 2,291517 | up | 0,014098 |
| DLG2 | 1170048 | 2,652251 | down | 0,038163 |  | HS.551042 | 6290154 | 2,292208 | up | 0,020715 |
| ABCA13 | 1780537 | 2,655062 | down | 0,009195 |  | PPP1R14B | 5290097 | 2,292664 | up | 0,002058 |
| ZNF648 | 1430343 | 2,656165 | down | 0,03204 |  | HS.568434 | 5050079 | 2,29548 | up | 0,042867 |
| CCDC33 | 830465 | 2,65756 | down | 0,048856 |  | LOC286528 | 7610332 | 2,298436 | up | 0,042935 |
| CTGF | 2640292 | 2,659833 | down | 0,011516 |  | HS.170701 | 6270064 | 2,301348 | up | 0,04188 |
| FCGBP | 130609 | 2,660825 | down | 0,029049 |  | PRKACG | 6510398 | 2,302044 | up | 0,01125 |
| RSPH10B | 4060360 | 2,66114 | down | 0,033836 |  | LMNB1 | 3420593 | 2,304412 | up | 0,034835 |
| RORB | 2370561 | 2,662375 | down | 0,029575 |  | LOC388532 | 2900470 | 2,307591 | up | 0,006189 |
| C2CD2L | 6520364 | 2,662537 | down | 0,004802 |  | ZFAND1 | 7380707 | 2,308215 | up | 0,029419 |
| C16ORF71 | 1090767 | 2,664131 | down | 0,032037 |  | LOC652128 | 6650020 | 2,30982 | up | 0,022742 |
| EFCAB6 | 3120093 | 2,671787 | down | 0,028203 |  | HS.563715 | 4290739 | 2,310338 | up | 0,01561 |
| NCALD | 520553 | 2,682396 | down | 0,003199 |  | PLK4 | 3190470 | 2,310516 | up | 0,038402 |
| NCALD | 60093 | 2,683965 | down | 0,004106 |  | LOC285176 | 290162 | 2,312214 | up | 0,019622 |
| PDE7B | 1240068 | 2,684702 | down | 0,007839 |  | HS.566820 | 5670056 | 2,31265 | up | 0,014135 |
| BCL9L | 5960471 | 2,685225 | down | 0,043119 |  | LOC652887 | 6370653 | 2,312913 | up | 0,031092 |
| IQGAP2 | 4220440 | 2,688261 | down | 0,035611 |  | LOC641996 | 4560368 | 2,31313 | up | 0,009098 |
| CD59 | 1410201 | 2,69076 | down | 0,010723 |  | LOC644739 | 270239 | 2,313666 | up | 0,026335 |
| TMC5 | 2140601 | 2,691619 | down | 0,011476 |  | LOC652552 | 1070154 | 2,316318 | up | 0,0097 |
| ICA1L | 7320541 | 2,693877 | down | 0,046249 |  | HS.577306 | 4610187 | 2,316471 | up | 0,009467 |
| COL5A2 | 4490292 | 2,696327 | down | 0,03302 |  | LOC644625 | 4810369 | 2,316755 | up | 0,005179 |
| DLEC1 | 5390131 | 2,697123 | down | 0,007829 |  | SIP1 | 7040609 | 2,317022 | up | 0,031409 |
| STEAP2 | 290292 | 2,697181 | down | 0,000305 |  | HS.579145 | 7040332 | 2,319457 | up | 0,048624 |
| HS.563240 | 4850288 | 2,698687 | down | 0,001202 |  | PRSS3 | 5080139 | 2,320578 | up | 0,033879 |
| LOC728343 | 2120273 | 2,699479 | down | 0,006507 |  | TEAD2 | 4670398 | 2,320984 | up | 0,004859 |
| C3ORF25 | 4210047 | 2,704545 | down | 0,021654 |  | MCM6 | 5690274 | 2,322253 | up | 0,007639 |
| CTGF | 5690687 | 2,708329 | down | 0,012684 |  | LRRC40 | 4780020 | 2,323387 | up | 0,020592 |
| KBTBD10 | 6960487 | 2,711243 | down | 0,006911 |  | RPLP0 | 1470349 | 2,324889 | up | 0,004205 |
| HPX | 2140196 | 2,711718 | down | 0,035238 |  | RPL22L1 | 1510743 | 2,330848 | up | 0,025465 |
| CXORF57 | 6580626 | 2,714968 | down | 0,038405 |  | HS.421532 | 7320465 | 2,33106 | up | 0,007125 |
| MGC20983 | 4260615 | 2,718014 | down | 0,02902 |  | AMY1A | 1740220 | 2,331262 | up | 0,012608 |
| ENPP5 | 540465 | 2,725413 | down | 0,01127 |  | HS.339592 | 4040154 | 2,331953 | up | 0,029715 |
| TSNAXIP1 | 2350221 | 2,733718 | down | 0,015001 |  | CDCA7 | 2070520 | 2,331987 | up | 0,040745 |
| LAMC2 | 2230594 | 2,737757 | down | 0,005378 |  | HS.542600 | 6180605 | 2,332366 | up | 0,004409 |
| GLB1L2 | 3940014 | 2,73777 | down | 0,009617 |  | TROAP | 4760646 | 2,336782 | up | 0,033835 |
| C9ORF21 | 1740075 | 2,741425 | down | 0,035544 |  | TRIM5 | 290373 | 2,337167 | up | 0,045289 |
| VWA3B | 7330615 | 2,744611 | down | 0,043594 |  | RPL29 | 4780433 | 2,337566 | up | 0,032958 |
| HYOU1 | 520189 | 2,747939 | down | 0,002152 |  | FYB | 5890414 | 2,338052 | up | 0,049881 |
| MAP1A | 4920202 | 2,748796 | down | 0,005667 |  | ZNF214 | 70154 | 2,339263 | up | 0,03 |
| C14ORF24 | 3990661 | 2,749284 | down | 0,03515 |  | LOC643070 | 7040121 | 2,340514 | up | 0,028144 |
| MGC50722 | 6860075 | 2,751611 | down | 0,018508 |  | CTHRC1 | 4860546 | 2,341232 | up | 0,03992 |
| C19ORF51 | 3520612 | 2,756091 | down | 0,008679 |  | CHPT1 | 2630687 | 2,343882 | up | 0,008693 |
| DNALI1 | 6200309 | 2,768005 | down | 0,019577 |  | CAPZA1 | 4610138 | 2,345571 | up | 0,038105 |
| C1ORF107 | 1300092 | 2,769137 | down | 0,025184 |  | PDCD10 | 6840274 | 2,348937 | up | 0,016579 |
| LOC92270 | 5490270 | 2,770217 | down | 7,27E-05 |  | LRRTM3 | 6380332 | 2,349422 | up | 0,036703 |
| TTLL1 | 1050521 | 2,770288 | down | 0,018406 |  | HS.575026 | 4830767 | 2,351931 | up | 0,005209 |
| CTDP1 | 4060167 | 2,771106 | down | 0,004861 |  | LOC643577 | 5670619 | 2,354343 | up | 0,000974 |
| KIF27 | 2000725 | 2,771991 | down | 0,04331 |  | SPIN2B | 770162 | 2,354513 | up | 0,004153 |
| HS.577681 | 7570646 | 2,775023 | down | 0,026361 |  | LOC643949 | 4290349 | 2,355042 | up | 0,003604 |
| POLR2C | 6960546 | 2,775027 | down | 0,027236 |  | HS.527758 | 4610452 | 2,35814 | up | 0,009252 |
| FLJ37464 | 5260019 | 2,775481 | down | 0,004209 |  | LOC643441 | 2060408 | 2,358888 | up | 0,00448 |
| SLC23A1 | 2690563 | 2,787675 | down | 0,014388 |  | TNFRSF4 | 650328 | 2,363974 | up | 0,025453 |
| RSPH10B | 5810324 | 2,795412 | down | 0,02268 |  | SUMO2 | 520133 | 2,366879 | up | 0,006119 |
| HHLA2 | 1450113 | 2,802754 | down | 0,033268 |  | HS.126473 | 2070398 | 2,367244 | up | 0,023925 |
| C2ORF65 | 5360300 | 2,806653 | down | 0,001389 |  | PDSS1 | 430192 | 2,369093 | up | 0,04574 |
| C20ORF96 | 130056 | 2,816036 | down | 0,022467 |  | LOC649030 | 240112 | 2,369185 | up | 0,014712 |
| HS.397465 | 3710437 | 2,82804 | down | 0,030863 |  | HS.564521 | 3370095 | 2,369916 | up | 0,006114 |
| SLC5A8 | 3520424 | 2,830021 | down | 0,040713 |  | HS.545255 | 2070647 | 2,370183 | up | 0,001208 |
| SETX | 4250601 | 2,830759 | down | 0,004672 |  | HS.583098 | 1030066 | 2,372174 | up | 0,002995 |
| PLEKHG7 | 3460632 | 2,833388 | down | 0,002606 |  | CHEK1 | 7200270 | 2,37242 | up | 0,01501 |
| UGCGL1 | 7650725 | 2,834382 | down | 0,0165 |  | HS.58068 | 840440 | 2,378701 | up | 0,02909 |
| WFS1 | 6590224 | 2,835793 | down | 0,000369 |  | HS.543242 | 2640739 | 2,380651 | up | 0,018199 |
| CNN1 | 4850630 | 2,83619 | down | 0,017448 |  | C20ORF27 | 2750647 | 2,382322 | up | 0,037716 |
| ATG9B | 2570204 | 2,853255 | down | 0,046281 |  | PI4K2B | 4180403 | 2,383097 | up | 0,015398 |
| DNAI2 | 5960685 | 2,853381 | down | 0,006119 |  | HNRPH1 | 6350343 | 2,38544 | up | 0,001708 |
| WDR86 | 7650068 | 2,855802 | down | 0,035833 |  | FAM26F | 6590646 | 2,386252 | up | 0,012467 |
| LOC730024 | 380097 | 2,855909 | down | 0,009564 |  | HAX1 | 6770494 | 2,387298 | up | 0,028824 |
| PRKAR2A | 4290170 | 2,861104 | down | 0,042297 |  | IKZF5 | 6480372 | 2,388303 | up | 0,031591 |
| FLJ16686 | 6330301 | 2,863726 | down | 0,014508 |  | LOC442283 | 6200435 | 2,390253 | up | 0,045125 |
| C18ORF1 | 3060390 | 2,870865 | down | 0,005605 |  | HS.531728 | 4280619 | 2,391437 | up | 0,0161 |
| LOC387885 | 6350538 | 2,871314 | down | 0,015478 |  | GPR101 | 7560332 | 2,392805 | up | 0,007691 |
| C10ORF63 | 1940112 | 2,877044 | down | 0,027975 |  | MED27 | 7100114 | 2,393673 | up | 0,027505 |
| MARK2 | 7320592 | 2,877357 | down | 0,027736 |  | ADFP | 460204 | 2,395007 | up | 0,028526 |
| RGS22 | 6760561 | 2,882035 | down | 0,017228 |  | HS.129392 | 7330239 | 2,397678 | up | 0,000805 |
| WDR38 | 2070441 | 2,886155 | down | 0,024291 |  | TSC22D1 | 380689 | 2,398191 | up | 0,036265 |
| DCDC5 | 3990630 | 2,888759 | down | 0,019623 |  | ASCC3 | 870040 | 2,4 | up | 0,011005 |
| PROC | 4810039 | 2,889907 | down | 0,018974 |  | ZWINT | 3800474 | 2,40641 | up | 0,039543 |
| SCAMP5 | 7510164 | 2,895739 | down | 0,007088 |  | STMN1 | 3460707 | 2,408766 | up | 0,001472 |
| MGC26718 | 1770392 | 2,900796 | down | 0,018744 |  | RAB32 | 2190128 | 2,408989 | up | 0,014389 |
| C13ORF30 | 3170288 | 2,911102 | down | 0,042592 |  | HS.571576 | 4860128 | 2,410313 | up | 0,006349 |
| CHST6 | 20445 | 2,930562 | down | 0,000602 |  | RPL39L | 6900672 | 2,41129 | up | 0,009317 |
| KIAA1688 | 4850300 | 2,937129 | down | 0,000763 |  | XYLT1 | 5820341 | 2,411524 | up | 0,020914 |
| YSK4 | 6350349 | 2,938737 | down | 0,010211 |  | ID1 | 670386 | 2,412973 | up | 0,006678 |
| GCLM | 4480053 | 2,939284 | down | 0,000531 |  | HS.197082 | 7040040 | 2,414908 | up | 0,000939 |
| MAPK10 | 6180521 | 2,940093 | down | 0,016943 |  | HS.439031 | 2070445 | 2,415577 | up | 0,024967 |
| OTUD5 | 60463 | 2,942595 | down | 0,004242 |  | CDCA5 | 130022 | 2,416565 | up | 0,019295 |
| SPEF1 | 2000037 | 2,942801 | down | 0,018947 |  | TNFRSF18 | 520066 | 2,4166 | up | 0,028902 |
| C1ORF87 | 7210477 | 2,948807 | down | 0,030317 |  | LOC651495 | 6200639 | 2,420072 | up | 0,010015 |
| FLJ21687 | 2100047 | 2,949165 | down | 0,009371 |  | HS.569204 | 4010246 | 2,420723 | up | 0,038996 |
| ELAVL1 | 1440215 | 2,962305 | down | 0,002865 |  | LOC440354 | 380754 | 2,422128 | up | 0,024651 |
| HS.547277 | 3780152 | 2,964525 | down | 0,042007 |  | LOC643615 | 5080554 | 2,427881 | up | 0,013187 |
| GRAMD2 | 5720619 | 2,971435 | down | 0,03569 |  | LOC646869 | 3940465 | 2,430403 | up | 0,003462 |
| PAPD5 | 520017 | 2,972103 | down | 0,002783 |  | TYROBP | 2370358 | 2,431572 | up | 0,006812 |
| SLC1A4 | 6350392 | 2,972641 | down | 0,00433 |  | LOC388588 | 1510017 | 2,43225 | up | 0,029829 |
| VSTM2L | 4390575 | 2,977154 | down | 0,001642 |  | PHLDA1 | 5900725 | 2,432531 | up | 0,045034 |
| PLEKHB1 | 2690133 | 2,996426 | down | 0,024539 |  | IMPACT | 6620435 | 2,435006 | up | 0,028978 |
| BIN3 | 1470291 | 2,996931 | down | 0,012258 |  | RPL23 | 380575 | 2,435446 | up | 0,001059 |
| C8ORF47 | 5690278 | 3,002461 | down | 0,020616 |  | HS.451341 | 1710128 | 2,43871 | up | 0,02718 |
| CTAGE5 | 7650504 | 3,005792 | down | 0,044326 |  | IL1RN | 2470601 | 2,438938 | up | 0,0061 |
| RABL5 | 5490484 | 3,008108 | down | 0,024699 |  | UBTD2 | 4760458 | 2,439911 | up | 0,00318 |
| DNHD2 | 1710253 | 3,013099 | down | 0,022748 |  | ACOT11 | 7400315 | 2,445707 | up | 0,002651 |
| MAPK8IP1 | 4150131 | 3,015463 | down | 0,008184 |  | CRABP2 | 3400296 | 2,447096 | up | 0,02383 |
| PLCH1 | 5910521 | 3,017069 | down | 0,014757 |  | HS.559999 | 4670465 | 2,447151 | up | 0,016402 |
| LOC387856 | 5550259 | 3,020616 | down | 0,007848 |  | RAD51AP1 | 4150196 | 2,449854 | up | 0,043331 |
| PCSK5 | 1740343 | 3,029387 | down | 0,006564 |  | MED21 | 130672 | 2,450104 | up | 0,0235 |
| SVOPL | 7330215 | 3,034186 | down | 0,029055 |  | LOC389787 | 160047 | 2,452322 | up | 0,001097 |
| WDR63 | 5560730 | 3,034299 | down | 0,048702 |  | HS.572030 | 730369 | 2,452403 | up | 0,040332 |
| CCDC135 | 1340471 | 3,036112 | down | 0,005121 |  | HS.552556 | 4480332 | 2,455071 | up | 0,021527 |
| PNLDC1 | 6940008 | 3,044897 | down | 0,0448 |  | CD58 | 5900594 | 2,457036 | up | 0,045675 |
| ADCY9 | 3060100 | 3,044985 | down | 0,017301 |  | CSAG3A | 4010095 | 2,458253 | up | 0,027825 |
| CYP2E1 | 670068 | 3,052195 | down | 0,019697 |  | ALG13 | 6660411 | 2,458283 | up | 0,012892 |
| TCTE1 | 780333 | 3,053249 | down | 0,007752 |  | HS.571249 | 2750239 | 2,458877 | up | 0,005756 |
| C1ORF88 | 1820692 | 3,057224 | down | 0,028706 |  | HS.577646 | 5820022 | 2,459532 | up | 0,023249 |
| KATNAL1 | 2470600 | 3,058289 | down | 0,006646 |  | HS.568690 | 5820026 | 2,460604 | up | 0,030383 |
| RNF150 | 3440133 | 3,060216 | down | 0,017694 |  | MS4A6A | 520360 | 2,460714 | up | 0,006449 |
| ATP6V0A4 | 5290243 | 3,073275 | down | 0,033379 |  | CCNG2 | 5360672 | 2,462715 | up | 0,030408 |
| RPL32 | 6960220 | 3,073661 | down | 0,00474 |  | DIAPH3 | 6660739 | 2,464648 | up | 0,001741 |
| PANX2 | 4070300 | 3,084721 | down | 0,020701 |  | RELB | 730440 | 2,465487 | up | 0,041071 |
| GRM7 | 4760670 | 3,085767 | down | 0,035754 |  | TMEM149 | 5670139 | 2,466467 | up | 0,045286 |
| ATP6V0A4 | 6110328 | 3,089287 | down | 0,021001 |  | HS.561747 | 3370037 | 2,468187 | up | 0,020807 |
| PTAFR | 6450093 | 3,093682 | down | 0,004506 |  | HS.144222 | 780397 | 2,469232 | up | 0,018251 |
| MIA | 6980767 | 3,094353 | down | 0,009696 |  | MMP28 | 830685 | 2,469756 | up | 0,001684 |
| HS.526959 | 5310131 | 3,100758 | down | 0,017896 |  | LOC134997 | 870537 | 2,470502 | up | 0,003043 |
| LOC401002 | 3180670 | 3,114976 | down | 0,036557 |  | HS.582159 | 870465 | 2,470695 | up | 0,041732 |
| LOH11CR2A | 5080592 | 3,119655 | down | 0,00894 |  | TTK | 5870725 | 2,47164 | up | 0,020812 |
| DNAJB13 | 1780309 | 3,131024 | down | 0,026734 |  | C19ORF40 | 4890364 | 2,472384 | up | 0,001829 |
| PACRG | 4210746 | 3,131551 | down | 0,039688 |  | HS.540797 | 1230398 | 2,4755 | up | 0,017528 |
| MDH1B | 870072 | 3,136305 | down | 0,03505 |  | PRR6 | 1030026 | 2,479888 | up | 0,019754 |
| SPAG1 | 4760131 | 3,13803 | down | 0,01743 |  | CHCHD8 | 4120025 | 2,480484 | up | 0,018718 |
| LRRC50 | 5550035 | 3,140012 | down | 0,010513 |  | FLJ14186 | 6450239 | 2,481462 | up | 0,010996 |
| LOC646100 | 2640767 | 3,152875 | down | 0,029774 |  | RDM1 | 5820056 | 2,484059 | up | 0,022429 |
| LOC283152 | 70523 | 3,159577 | down | 0,009986 |  | PNRC2 | 1010202 | 2,486844 | up | 0,000263 |
| LOC285908 | 1660762 | 3,159657 | down | 0,033141 |  | PRC1 | 2070494 | 2,49031 | up | 0,006032 |
| ASTN2 | 360025 | 3,159914 | down | 0,034115 |  | HS.350952 | 7040050 | 2,490365 | up | 0,012626 |
| RAB36 | 7320129 | 3,167089 | down | 0,002136 |  | LOC728739 | 6350048 | 2,494072 | up | 0,040258 |
| STEAP2 | 870242 | 3,170437 | down | 0,000484 |  | HS.575015 | 3460445 | 2,494876 | up | 0,005604 |
| SULT1A2 | 6400326 | 3,173161 | down | 0,008239 |  | COMMD8 | 650148 | 2,502927 | up | 0,011245 |
| HS.298873 | 3830661 | 3,182645 | down | 0,022178 |  | DCK | 3800348 | 2,504604 | up | 0,003745 |
| HS.551143 | 6960075 | 3,183893 | down | 0,037987 |  | HS.566024 | 3610441 | 2,505915 | up | 0,017887 |
| RRAD | 7200639 | 3,190707 | down | 0,012479 |  | MAGEA12 | 4830739 | 2,506048 | up | 0,038054 |
| HS.13438 | 1190674 | 3,193628 | down | 0,010589 |  | HS.540255 | 7650465 | 2,507544 | up | 0,045454 |
| C6ORF224 | 5270670 | 3,197714 | down | 0,009996 |  | HS.542001 | 4010538 | 2,50846 | up | 0,006979 |
| GP2 | 1740630 | 3,204896 | down | 0,043915 |  | HS.546047 | 4210441 | 2,509713 | up | 0,006982 |
| ABCC6 | 3060279 | 3,205424 | down | 0,026355 |  | HS.542949 | 1450037 | 2,519495 | up | 4,68E-07 |
| ATXN1 | 520601 | 3,206385 | down | 0,015332 |  | HS.571403 | 2060332 | 2,520304 | up | 0,032242 |
| MAP6 | 1260296 | 3,210855 | down | 0,007367 |  | HMGB2 | 5900482 | 2,52258 | up | 0,010805 |
| AQP7P2 | 5720246 | 3,210973 | down | 0,021869 |  | LOC644150 | 6200020 | 2,52365 | up | 0,010944 |
| LRRC18 | 2900221 | 3,214613 | down | 0,027128 |  | GMNN | 4830373 | 2,52601 | up | 0,007523 |
| IPP | 2100519 | 3,21932 | down | 0,011513 |  | GJB2 | 5260095 | 2,526287 | up | 0,009912 |
| WDR93 | 4860044 | 3,23142 | down | 0,01803 |  | HS.581912 | 6450019 | 2,528677 | up | 0,01266 |
| SERPINB7 | 450463 | 3,236747 | down | 0,027154 |  | HS.577713 | 6660736 | 2,537647 | up | 0,001095 |
| SPAG17 | 2450653 | 3,244371 | down | 0,010919 |  | HS.548645 | 3780538 | 2,538244 | up | 0,013863 |
| WDR78 | 2360386 | 3,253022 | down | 0,018728 |  | HS.539278 | 1050601 | 2,539284 | up | 0,012914 |
| PROM1 | 7400452 | 3,254551 | down | 0,025467 |  | C12ORF48 | 1400743 | 2,539657 | up | 0,002898 |
| ADHFE1 | 3890598 | 3,258045 | down | 0,000748 |  | NAE1 | 6510619 | 2,539907 | up | 0,046351 |
| CYP2U1 | 1470224 | 3,258599 | down | 0,010191 |  | SLBP | 4280603 | 2,541255 | up | 0,007164 |
| TSPYL4 | 610521 | 3,259559 | down | 0,016666 |  | LOC730746 | 2260025 | 2,541821 | up | 0,016799 |
| LOC146177 | 3370168 | 3,264574 | down | 0,010072 |  | RPL14 | 2140753 | 2,544245 | up | 0,009267 |
| DNAH12L | 6110707 | 3,264584 | down | 0,012452 |  | HS.543437 | 2690162 | 2,54878 | up | 0,009548 |
| KNDC1 | 4830594 | 3,268394 | down | 0,033915 |  | NUDCD2 | 670195 | 2,54896 | up | 0,018216 |
| PLCB2 | 2060451 | 3,278618 | down | 0,005878 |  | LOC646900 | 3460162 | 2,551717 | up | 0,029788 |
| UBQLNL | 840360 | 3,278702 | down | 0,007502 |  | DTL | 5570296 | 2,55375 | up | 0,010255 |
| HS.161238 | 430750 | 3,287224 | down | 0,008532 |  | ANXA8 | 1400711 | 2,556929 | up | 0,009379 |
| DNHD1 | 3290392 | 3,287788 | down | 0,026271 |  | DMBT1 | 4880458 | 2,557757 | up | 0,035332 |
| AGBL2 | 4890338 | 3,291546 | down | 0,031872 |  | TSPAN12 | 3830193 | 2,558306 | up | 0,031762 |
| C10ORF92 | 3930672 | 3,297435 | down | 0,010501 |  | COMMD10 | 1740392 | 2,560184 | up | 0,024671 |
| DNAH1 | 4900612 | 3,301499 | down | 0,002095 |  | LOC642656 | 2100020 | 2,560941 | up | 0,016861 |
| SCARF2 | 5390703 | 3,306908 | down | 0,003731 |  | CMTM3 | 6020746 | 2,562102 | up | 0,016906 |
| C1ORF175 | 4540070 | 3,314948 | down | 0,005574 |  | HS.557901 | 6370154 | 2,564199 | up | 0,008911 |
| LRRC61 | 3830047 | 3,315536 | down | 0,004303 |  | RNASE1 | 1090307 | 2,564518 | up | 0,034558 |
| OS9 | 1030593 | 3,31984 | down | 0,005563 |  | HS.533020 | 1400064 | 2,56615 | up | 0,049508 |
| SLC35A2 | 4890224 | 3,321158 | down | 0,029338 |  | LOC646849 | 840577 | 2,568719 | up | 0,03697 |
| DNAL1 | 6100682 | 3,335572 | down | 0,007245 |  | CCDC90B | 2750192 | 2,569156 | up | 0,039903 |
| PTPRT | 5220114 | 3,336297 | down | 0,015872 |  | ALG13 | 730520 | 2,569157 | up | 0,02146 |
| LASS4 | 6590139 | 3,341908 | down | 0,004501 |  | HS.125626 | 6760020 | 2,57138 | up | 0,011339 |
| TTC18 | 6760541 | 3,349182 | down | 0,007495 |  | C12ORF45 | 7510608 | 2,571554 | up | 0,017027 |
| EFCAB6 | 2260494 | 3,352687 | down | 0,015366 |  | TMEM159 | 7560575 | 2,572971 | up | 0,031819 |
| NWD1 | 6280592 | 3,354395 | down | 0,003153 |  | LOC650454 | 520020 | 2,573125 | up | 0,00533 |
| TTC25 | 7550021 | 3,365454 | down | 0,028379 |  | BUB1 | 2070224 | 2,573623 | up | 0,039106 |
| CSPP1 | 6860709 | 3,366495 | down | 0,006048 |  | ZNF658B | 4780593 | 2,576439 | up | 0,002377 |
| SPAG8 | 430451 | 3,366541 | down | 0,020103 |  | ADA | 7210192 | 2,577757 | up | 0,008391 |
| MAPK15 | 6480451 | 3,368027 | down | 0,004388 |  | MYO19 | 5910020 | 2,579701 | up | 0,012836 |
| HS.150067 | 70288 | 3,369082 | down | 0,001573 |  | DST | 2940129 | 2,583276 | up | 0,029244 |
| TGM3 | 4920075 | 3,369128 | down | 0,026502 |  | HS.540472 | 2000408 | 2,586338 | up | 0,002463 |
| DNAH7 | 6100661 | 3,377328 | down | 0,004465 |  | MGC40489 | 3420400 | 2,586502 | up | 0,02825 |
| CABYR | 6250131 | 3,380918 | down | 0,017186 |  | NUP62 | 2510279 | 2,5867 | up | 0,010204 |
| BAIAP3 | 780719 | 3,388787 | down | 0,00302 |  | HS.545899 | 6330020 | 2,587929 | up | 0,027561 |
| LPIN2 | 510224 | 3,402575 | down | 0,005025 |  | TNFRSF18 | 840386 | 2,588707 | up | 0,005563 |
| C11ORF16 | 670543 | 3,409804 | down | 0,000121 |  | EVI2B | 7050152 | 2,595955 | up | 0,038375 |
| PTPRT | 4780138 | 3,410308 | down | 0,011673 |  | BLM | 2450717 | 2,59704 | up | 0,001465 |
| WFDC6 | 5420296 | 3,411129 | down | 0,047156 |  | ATMIN | 6620600 | 2,600051 | up | 0,033952 |
| QSOX1 | 4210634 | 3,414513 | down | 0,003299 |  | HS.541025 | 2600653 | 2,600177 | up | 0,010795 |
| LOC285359 | 3180347 | 3,415813 | down | 0,015044 |  | LOC646746 | 3370446 | 2,600698 | up | 0,001549 |
| C16ORF35 | 4150563 | 3,428664 | down | 0,003605 |  | HS.200085 | 4670332 | 2,600751 | up | 0,021044 |
| CAPS | 2650474 | 3,434655 | down | 0,013051 |  | C3ORF28 | 5310736 | 2,601382 | up | 0,000187 |
| LRGUK | 1580554 | 3,439464 | down | 0,014618 |  | ID3 | 7570324 | 2,605254 | up | 0,014179 |
| WDR78 | 2650754 | 3,441324 | down | 0,035798 |  | DBP | 7050458 | 2,606511 | up | 0,022414 |
| NEK11 | 4010022 | 3,442901 | down | 0,013441 |  | GALNTL1 | 240746 | 2,609508 | up | 0,006568 |
| PRG2 | 1580195 | 3,488399 | down | 0,004929 |  | HS.545755 | 290020 | 2,610618 | up | 0,010495 |
| VNN3 | 4010133 | 3,489604 | down | 0,010406 |  | RFC4 | 7210435 | 2,613212 | up | 0,025211 |
| FAM134B | 270114 | 3,497302 | down | 0,00263 |  | LOC645460 | 1030041 | 2,617575 | up | 0,03919 |
| C6ORF199 | 1190017 | 3,500289 | down | 0,000658 |  | G3BP1 | 5720300 | 2,619904 | up | 0,033082 |
| CCDC114 | 7210482 | 3,507234 | down | 0,017073 |  | FAM49B | 2630703 | 2,621547 | up | 0,026116 |
| FLJ90086 | 7150575 | 3,509527 | down | 0,0041 |  | HS.508889 | 2760307 | 2,625318 | up | 0,043766 |
| NUMA1 | 4120338 | 3,515761 | down | 0,014423 |  | TNFSF13B | 4900435 | 2,626795 | up | 0,020532 |
| NEK11 | 6480427 | 3,521987 | down | 0,014365 |  | DLK2 | 1070279 | 2,628638 | up | 0,009658 |
| SHANK2 | 4220209 | 3,522579 | down | 0,000492 |  | LOC648659 | 630669 | 2,629803 | up | 0,013257 |
| WFDC2 | 4050136 | 3,531178 | down | 0,044607 |  | LOC642616 | 770397 | 2,630335 | up | 0,003549 |
| MGC18216 | 6400639 | 3,553782 | down | 0,014702 |  | LOC399942 | 1740673 | 2,631315 | up | 0,044621 |
| C20ORF195 | 7040753 | 3,564546 | down | 0,0351 |  | LOC143543 | 3520470 | 2,635055 | up | 0,024409 |
| ACSBG1 | 2480730 | 3,587684 | down | 0,017644 |  | LOC644841 | 5290020 | 2,63529 | up | 0,02615 |
| C9ORF117 | 4260280 | 3,622016 | down | 0,006153 |  | PCNA | 6900079 | 2,636175 | up | 0,001705 |
| PRRT3 | 4070180 | 3,624087 | down | 0,00192 |  | FAM89A | 7400747 | 2,636592 | up | 0,044195 |
| SERPINI2 | 2680370 | 3,638912 | down | 0,002381 |  | LOC647281 | 1400255 | 2,639972 | up | 0,004379 |
| CES4 | 270575 | 3,656064 | down | 0,047147 |  | FSCN1 | 4560328 | 2,640973 | up | 0,049653 |
| ENAH | 3170132 | 3,668708 | down | 0,018034 |  | HS.564597 | 6770246 | 2,642085 | up | 0,001834 |
| CACNG6 | 5080180 | 3,715459 | down | 0,026025 |  | LOC285423 | 160056 | 2,654255 | up | 0,046816 |
| NEK10 | 240047 | 3,720847 | down | 0,004554 |  | RPL39L | 6060719 | 2,665276 | up | 0,039499 |
| PKN1 | 2360474 | 3,743072 | down | 0,006367 |  | CDKN3 | 5260014 | 2,666277 | up | 0,007165 |
| UBXD5 | 7610594 | 3,750186 | down | 0,019534 |  | C13ORF34 | 580719 | 2,675773 | up | 0,008488 |
| C9ORF98 | 6330358 | 3,765339 | down | 0,021159 |  | LOC644029 | 160575 | 2,679604 | up | 0,004522 |
| CROCC | 610424 | 3,778325 | down | 0,000342 |  | HS.543887 | 6840477 | 2,686704 | up | 0,020905 |
| CFTR | 5870131 | 3,783698 | down | 0,005948 |  | DKK2 | 2140441 | 2,688875 | up | 0,023512 |
| C3 | 4860494 | 3,794597 | down | 0,013493 |  | LOC388397 | 4880037 | 2,69253 | up | 0,005352 |
| ENO2 | 50402 | 3,82222 | down | 0,002968 |  | LOC649946 | 6270605 | 2,693251 | up | 0,000681 |
| LOC387856 | 4850189 | 3,824385 | down | 0,010286 |  | PDCD4 | 3130168 | 2,697671 | up | 0,047975 |
| DLEC1 | 7200035 | 3,855943 | down | 0,002565 |  | TPRKB | 6510044 | 2,701464 | up | 0,020717 |
| NXF2 | 4860333 | 3,856611 | down | 0,03196 |  | RPL9 | 4640095 | 2,701578 | up | 0,045462 |
| RHPN1 | 6590709 | 3,862965 | down | 0,002138 |  | RPLP1 | 2690561 | 2,703574 | up | 0,005313 |
| DKFZP586H2123 | 7320669 | 3,870434 | down | 0,019845 |  | CDCA3 | 6370474 | 2,705347 | up | 0,012244 |
| IFT88 | 4490253 | 3,872654 | down | 0,000316 |  | HS.560095 | 730239 | 2,715276 | up | 0,010602 |
| C1QTNF5 | 7320204 | 3,874789 | down | 0,025073 |  | LOC220433 | 6200747 | 2,717008 | up | 0,000987 |
| THRA | 3830138 | 3,875452 | down | 0,00166 |  | FAM83A | 3390692 | 2,720099 | up | 0,033146 |
| WDR66 | 4260286 | 3,885426 | down | 0,006775 |  | RPL7 | 2680082 | 2,723438 | up | 0,003925 |
| TUB | 4560072 | 3,889468 | down | 0,004881 |  | FAM89A | 6280048 | 2,724789 | up | 0,033495 |
| LOC222967 | 2030440 | 3,892602 | down | 0,008774 |  | PKMYT1 | 2510678 | 2,727237 | up | 0,011637 |
| C20ORF26 | 6110017 | 3,91404 | down | 0,002447 |  | ATP5S | 2120100 | 2,72979 | up | 0,027835 |
| SPEF2 | 5130528 | 3,914692 | down | 0,002972 |  | C6ORF211 | 3710465 | 2,733075 | up | 0,010752 |
| CSMD1 | 1010189 | 3,922024 | down | 0,005373 |  | GTF2IRD2B | 6200746 | 2,736995 | up | 0,001146 |
| C10ORF115 | 110152 | 3,922761 | down | 0,032163 |  | C20ORF100 | 1400601 | 2,737613 | up | 0,032517 |
| C1ORF173 | 4780475 | 3,928444 | down | 0,032383 |  | CENPK | 5700086 | 2,740421 | up | 0,005653 |
| FZD8 | 3290301 | 3,953607 | down | 0,002051 |  | HCG22 | 840398 | 2,743072 | up | 0,008937 |
| PZP | 2760711 | 3,957227 | down | 0,012192 |  | LOC728242 | 4850020 | 2,747375 | up | 0,010877 |
| ABCA2 | 2320243 | 3,965287 | down | 0,002655 |  | ZNF527 | 1710465 | 2,748489 | up | 0,01544 |
| HS.249972 | 2650725 | 3,980439 | down | 0,005428 |  | ZNF566 | 6650056 | 2,749418 | up | 0,010208 |
| TMEM45B | 430541 | 3,984178 | down | 0,001206 |  | HS.535456 | 1400671 | 2,749622 | up | 0,001866 |
| LOC619208 | 2750402 | 3,992654 | down | 0,003302 |  | RBM7 | 1340202 | 2,752902 | up | 0,020176 |
| DYNC2H1 | 4220215 | 4,026183 | down | 0,0058 |  | NMD3 | 4010048 | 2,753904 | up | 0,018183 |
| HYDIN | 1170484 | 4,076131 | down | 0,006027 |  | CCNB2 | 5360070 | 2,754705 | up | 0,037018 |
| FCGBP | 130463 | 4,087641 | down | 0,005543 |  | HS.579678 | 4900056 | 2,76356 | up | 0,0039 |
| FLJ23834 | 5220139 | 4,098799 | down | 0,015464 |  | LOC731486 | 2490328 | 2,764984 | up | 0,024788 |
| C2ORF62 | 2680564 | 4,111105 | down | 0,011118 |  | LOC650739 | 7000056 | 2,766869 | up | 0,004334 |
| MORN3 | 6040307 | 4,138602 | down | 0,034949 |  | MPP6 | 3370280 | 2,767595 | up | 0,003428 |
| CYP2C8 | 2100711 | 4,139114 | down | 0,005531 |  | GZMK | 1260482 | 2,772199 | up | 0,013168 |
| CDKL2 | 2630280 | 4,15399 | down | 0,000485 |  | FAM49B | 7160079 | 2,773186 | up | 0,00035 |
| TEKT3 | 2030176 | 4,194599 | down | 0,010767 |  | TMEM16A | 6420025 | 2,780844 | up | 0,037874 |
| PROS1 | 6420008 | 4,216316 | down | 0,008639 |  | NRM | 5670059 | 2,785292 | up | 0,017416 |
| ALDH3B1 | 1850440 | 4,22533 | down | 0,001495 |  | HS.551128 | 5900246 | 2,785377 | up | 0,009282 |
| MORN1 | 2030564 | 4,239786 | down | 0,007488 |  | SLC9A4 | 3130360 | 2,789639 | up | 0,036869 |
| HS.427242 | 7210161 | 4,295359 | down | 0,01222 |  | TYMS | 5910364 | 2,792096 | up | 0,021816 |
| PARC | 6580491 | 4,299237 | down | 0,002549 |  | LST1 | 650112 | 2,795292 | up | 0,0238 |
| C2ORF40 | 3140113 | 4,331877 | down | 0,033392 |  | GLRX2 | 6980274 | 2,808733 | up | 0,012144 |
| LOC649864 | 130594 | 4,333318 | down | 0,008344 |  | WASPIP | 5700670 | 2,810177 | up | 0,011298 |
| HS.529631 | 7200286 | 4,348695 | down | 0,001513 |  | LOC644310 | 3850452 | 2,812456 | up | 0,007434 |
| HYDIN | 7550360 | 4,350644 | down | 0,000905 |  | ZCCHC16 | 5700162 | 2,813119 | up | 0,019382 |
| LOC645799 | 4920187 | 4,365395 | down | 0,017733 |  | WNT4 | 6840202 | 2,813807 | up | 0,036081 |
| DNAH2 | 6860689 | 4,368558 | down | 0,003188 |  | MRPL10 | 6180154 | 2,819538 | up | 0,01239 |
| CES1 | 2680056 | 4,375245 | down | 0,004444 |  | HS.541762 | 1780121 | 2,822727 | up | 0,000529 |
| CCDC37 | 7570682 | 4,402967 | down | 0,008948 |  | LOC347376 | 3940364 | 2,825347 | up | 0,000771 |
| DNAH9 | 6400255 | 4,403091 | down | 0,002619 |  | CCBE1 | 6560465 | 2,825401 | up | 0,008752 |
| KIAA1683 | 3850754 | 4,412383 | down | 0,007209 |  | RPS26L | 5890730 | 2,835035 | up | 5,62E-05 |
| ZFHX2 | 2370484 | 4,457166 | down | 0,000222 |  | HS.576473 | 7550020 | 2,858097 | up | 0,004414 |
| MPL | 5050086 | 4,492312 | down | 0,000644 |  | MGC40489 | 4670605 | 2,867809 | up | 0,004187 |
| DZIP1L | 1990601 | 4,496159 | down | 0,000142 |  | HS.573434 | 1430022 | 2,87294 | up | 0,009332 |
| TTLL10 | 3140161 | 4,507662 | down | 0,001304 |  | APH1A | 7550364 | 2,876247 | up | 0,005111 |
| CACNG6 | 1990482 | 4,517551 | down | 0,00043 |  | C12ORF31 | 2940546 | 2,879136 | up | 0,011299 |
| TMEM190 | 6200537 | 4,519313 | down | 0,015101 |  | HNRPC | 1500201 | 2,888187 | up | 0,034965 |
| CD59 | 1430240 | 4,572104 | down | 0,001095 |  | HS.549551 | 6840397 | 2,890493 | up | 0,006438 |
| LOC389118 | 5260482 | 4,573442 | down | 0,006759 |  | AURKA | 4730605 | 2,891524 | up | 0,024145 |
| BEST4 | 3370180 | 4,638632 | down | 0,011306 |  | PTTG1 | 1510291 | 2,900304 | up | 0,003772 |
| RNF190 | 5860446 | 4,663751 | down | 0,00499 |  | LOC641848 | 2640255 | 2,90296 | up | 0,011512 |
| C9ORF68 | 5890332 | 4,711139 | down | 0,004812 |  | EEF1B2 | 3780246 | 2,905711 | up | 0,031976 |
| HS.570821 | 6110603 | 4,72936 | down | 0,001766 |  | LOC651894 | 2710451 | 2,908917 | up | 0,001949 |
| MUC15 | 3850451 | 4,765177 | down | 0,000738 |  | VPS26 | 4010689 | 2,910768 | up | 0,016681 |
| C1ORF110 | 7050215 | 4,822424 | down | 0,000504 |  | FAM54A | 4220544 | 2,913136 | up | 0,000804 |
| DNAH3 | 2680059 | 5,012676 | down | 0,002668 |  | PLA2G7 | 3390438 | 2,914993 | up | 0,038997 |
| LOC643293 | 5310347 | 5,037606 | down | 7,96E-05 |  | LOC647037 | 5700709 | 2,916097 | up | 0,04107 |
| FAM92B | 1190152 | 5,161243 | down | 0,002137 |  | PTTG1 | 10414 | 2,917897 | up | 0,006018 |
| HS.545615 | 3060358 | 5,196993 | down | 0,032742 |  | NUDCD2 | 610376 | 2,924195 | up | 0,042843 |
| MGC33556 | 1430634 | 5,276831 | down | 0,003117 |  | HS.85989 | 2570056 | 2,9293 | up | 0,007158 |
| HGD | 2100358 | 5,498459 | down | 0,006402 |  | LOC644380 | 7550605 | 2,931329 | up | 0,009798 |
| LOC645799 | 2120646 | 5,547714 | down | 0,003501 |  | ECHDC1 | 7650053 | 2,940011 | up | 0,044331 |
| CXXC4 | 510192 | 5,609991 | down | 2,28E-05 |  | C5ORF13 | 940471 | 2,954726 | up | 0,023592 |
| SLC6A16 | 620707 | 5,675763 | down | 0,001853 |  | HMMR | 4050400 | 2,955086 | up | 0,042383 |
| DNAH5 | 2350554 | 5,692593 | down | 0,003368 |  | VKORC1 | 6450546 | 2,963459 | up | 0,007749 |
| KIAA0319 | 4920112 | 5,781835 | down | 0,002536 |  | KIFC1 | 5090095 | 2,965692 | up | 0,002857 |
| HS.127963 | 4040136 | 5,829488 | down | 0,000639 |  | HS.571181 | 4540731 | 2,976705 | up | 0,018015 |
| HS.531817 | 7160176 | 6,27119 | down | 0,002031 |  | LOC648684 | 6590020 | 2,979308 | up | 0,02458 |
| DNAH11 | 7330360 | 6,322539 | down | 0,004405 |  | LOC645466 | 1010458 | 2,988287 | up | 0,019745 |
| SLC7A2 | 3710154 | 6,649861 | down | 0,0003 |  | LOC389672 | 3180131 | 2,988461 | up | 0,004786 |
| KIAA1751 | 1510463 | 7,892708 | down | 0,00095 |  | LOC342897 | 3840148 | 2,995559 | up | 0,046535 |
| OXTR | 7050768 | 8,899006 | down | 0,006246 |  | NMD3 | 5360204 | 2,998491 | up | 0,01799 |
| ALB | 650431 | 12,35059 | down | 0,018062 |  | TMEM167 | 3850224 | 3,000801 | up | 0,003552 |
| RYR3 | 430253 | 13,83864 | down | 0,000787 |  | LOC728973 | 3870470 | 3,014725 | up | 0,001358 |
| HLA-A29,1 | 5080692 | 83,63067 | down | 0,001407 |  | PRSS27 | 7210600 | 3,014746 | up | 0,000712 |
|  |  |  |  |  |  | C18ORF56 | 1450682 | 3,020253 | up | 0,023241 |
|  |  |  |  |  |  | NUSAP1 | 1500553 | 3,026574 | up | 0,004753 |
|  |  |  |  |  |  | LOC644482 | 150292 | 3,032751 | up | 0,01253 |
|  |  |  |  |  |  | CKS2 | 780528 | 3,034463 | up | 0,000731 |
|  |  |  |  |  |  | CKS2 | 6200468 | 3,036278 | up | 0,001553 |
|  |  |  |  |  |  | MMP10 | 150180 | 3,04068 | up | 0,034869 |
|  |  |  |  |  |  | UHRF1 | 2940110 | 3,042475 | up | 0,000315 |
|  |  |  |  |  |  | ANKDD1A | 5890564 | 3,049458 | up | 0,03668 |
|  |  |  |  |  |  | EVI2A | 4590224 | 3,052574 | up | 0,026483 |
|  |  |  |  |  |  | RMND1 | 3360220 | 3,065317 | up | 0,015469 |
|  |  |  |  |  |  | RPL29 | 2450167 | 3,069007 | up | 0,003831 |
|  |  |  |  |  |  | DHDH | 6770630 | 3,082407 | up | 0,004195 |
|  |  |  |  |  |  | GPR87 | 4180181 | 3,086987 | up | 0,009015 |
|  |  |  |  |  |  | HMGB1 | 1820600 | 3,087613 | up | 0,03493 |
|  |  |  |  |  |  | FAM46B | 4180324 | 3,095732 | up | 0,032238 |
|  |  |  |  |  |  | MCTS1 | 1690025 | 3,102661 | up | 0,01498 |
|  |  |  |  |  |  | ADAMDEC1 | 2470184 | 3,106379 | up | 0,012019 |
|  |  |  |  |  |  | TPD52L1 | 7150349 | 3,108623 | up | 0,039483 |
|  |  |  |  |  |  | DSC2 | 2710400 | 3,109318 | up | 0,015093 |
|  |  |  |  |  |  | C7ORF28B | 50689 | 3,109517 | up | 0,046129 |
|  |  |  |  |  |  | IFP38 | 5290482 | 3,109842 | up | 0,000862 |
|  |  |  |  |  |  | KCNS3 | 540204 | 3,111803 | up | 0,018271 |
|  |  |  |  |  |  | CEP55 | 7510709 | 3,115749 | up | 0,032531 |
|  |  |  |  |  |  | EXO1 | 1770646 | 3,141219 | up | 0,005256 |
|  |  |  |  |  |  | PMAIP1 | 6020598 | 3,142931 | up | 0,014207 |
|  |  |  |  |  |  | PLA2G4A | 4730360 | 3,15142 | up | 0,001279 |
|  |  |  |  |  |  | TOP2A | 3990619 | 3,180671 | up | 0,04494 |
|  |  |  |  |  |  | MAD2L1 | 870546 | 3,181268 | up | 0,010231 |
|  |  |  |  |  |  | LOC648827 | 2680037 | 3,184769 | up | 0,019642 |
|  |  |  |  |  |  | FABP5 | 4150048 | 3,188683 | up | 0,016303 |
|  |  |  |  |  |  | C7ORF11 | 2900047 | 3,201285 | up | 0,012659 |
|  |  |  |  |  |  | LOC653232 | 5050541 | 3,210611 | up | 0,001369 |
|  |  |  |  |  |  | C7ORF28B | 6760670 | 3,212907 | up | 0,019872 |
|  |  |  |  |  |  | HS.541602 | 1240056 | 3,21332 | up | 0,015287 |
|  |  |  |  |  |  | LOC643007 | 7650220 | 3,220597 | up | 0,027072 |
|  |  |  |  |  |  | PDPN | 3420288 | 3,225845 | up | 0,048709 |
|  |  |  |  |  |  | LOC441377 | 830484 | 3,260879 | up | 0,000237 |
|  |  |  |  |  |  | GNG4 | 4050671 | 3,26229 | up | 0,010625 |
|  |  |  |  |  |  | UBE2C | 4260368 | 3,274615 | up | 0,019256 |
|  |  |  |  |  |  | LOC642989 | 6280446 | 3,285336 | up | 0,001523 |
|  |  |  |  |  |  | DNASE1L3 | 290739 | 3,301242 | up | 0,036038 |
|  |  |  |  |  |  | ICAM4 | 730554 | 3,302833 | up | 0,001738 |
|  |  |  |  |  |  | LY86 | 6840408 | 3,312323 | up | 0,002757 |
|  |  |  |  |  |  | LOC346950 | 3930707 | 3,315732 | up | 0,004744 |
|  |  |  |  |  |  | LOC440926 | 2360136 | 3,319802 | up | 0,003528 |
|  |  |  |  |  |  | PTPLA | 730221 | 3,338682 | up | 0,011267 |
|  |  |  |  |  |  | UQCRH | 6960735 | 3,34443 | up | 0,001019 |
|  |  |  |  |  |  | LOC651202 | 6020066 | 3,363637 | up | 0,000497 |
|  |  |  |  |  |  | PTMA | 460386 | 3,375706 | up | 0,000315 |
|  |  |  |  |  |  | LOC645018 | 7320196 | 3,386552 | up | 0,002622 |
|  |  |  |  |  |  | LOC647673 | 4220307 | 3,388853 | up | 0,002738 |
|  |  |  |  |  |  | U2AF1 | 3930241 | 3,394796 | up | 0,004665 |
|  |  |  |  |  |  | KIAA0101 | 5090754 | 3,411974 | up | 0,000511 |
|  |  |  |  |  |  | SPC24 | 5910349 | 3,418563 | up | 0,020183 |
|  |  |  |  |  |  | CENPA | 2600392 | 3,421464 | up | 0,000354 |
|  |  |  |  |  |  | PADI3 | 3890601 | 3,425994 | up | 0,040327 |
|  |  |  |  |  |  | SRGN | 7550484 | 3,431644 | up | 0,009442 |
|  |  |  |  |  |  | SNHG10 | 830594 | 3,431936 | up | 0,032863 |
|  |  |  |  |  |  | C7ORF28A | 3130477 | 3,462847 | up | 0,016943 |
|  |  |  |  |  |  | COPS2 | 4890195 | 3,479432 | up | 0,004959 |
|  |  |  |  |  |  | MS4A6A | 1770152 | 3,486726 | up | 0,044342 |
|  |  |  |  |  |  | PMAIP1 | 2750367 | 3,508389 | up | 0,026093 |
|  |  |  |  |  |  | DST | 5560241 | 3,511753 | up | 0,03581 |
|  |  |  |  |  |  | LOC402057 | 3610475 | 3,545203 | up | 0,003349 |
|  |  |  |  |  |  | FABP5 | 5260047 | 3,569892 | up | 0,015336 |
|  |  |  |  |  |  | SRGN | 360500 | 3,570247 | up | 0,033155 |
|  |  |  |  |  |  | LOC651453 | 4900402 | 3,580185 | up | 0,000277 |
|  |  |  |  |  |  | DUSP11 | 4060523 | 3,623903 | up | 0,0004 |
|  |  |  |  |  |  | CDC2 | 1050706 | 3,644996 | up | 0,011445 |
|  |  |  |  |  |  | LOC648343 | 3370201 | 3,65956 | up | 0,001016 |
|  |  |  |  |  |  | MGC39900 | 2000154 | 3,688653 | up | 0,011068 |
|  |  |  |  |  |  | LOC647104 | 6280195 | 3,701692 | up | 0,001069 |
|  |  |  |  |  |  | AP2A1 | 5890465 | 3,711945 | up | 2,49E-05 |
|  |  |  |  |  |  | LOC643870 | 3400762 | 3,729183 | up | 0,001612 |
|  |  |  |  |  |  | OIP5 | 7000161 | 3,802264 | up | 0,003781 |
|  |  |  |  |  |  | C6ORF173 | 670703 | 3,815007 | up | 0,002626 |
|  |  |  |  |  |  | MELK | 160097 | 3,842004 | up | 0,019738 |
|  |  |  |  |  |  | GPC3 | 1340593 | 3,84563 | up | 0,00444 |
|  |  |  |  |  |  | LYZ | 3710504 | 3,856075 | up | 0,039619 |
|  |  |  |  |  |  | FGFBP1 | 7650441 | 3,859391 | up | 0,041292 |
|  |  |  |  |  |  | TUBA3D | 3930132 | 3,862404 | up | 0,000102 |
|  |  |  |  |  |  | S100A2 | 2970017 | 3,879618 | up | 0,00854 |
|  |  |  |  |  |  | IRF8 | 150072 | 3,904401 | up | 0,022581 |
|  |  |  |  |  |  | LOC388344 | 7400047 | 3,918175 | up | 0,014 |
|  |  |  |  |  |  | LSM5 | 6650133 | 3,983964 | up | 0,011643 |
|  |  |  |  |  |  | LY6D | 5270246 | 4,043026 | up | 0,009936 |
|  |  |  |  |  |  | UBE2C | 5310471 | 4,049859 | up | 0,007051 |
|  |  |  |  |  |  | CHPT1 | 2260600 | 4,065369 | up | 0,008014 |
|  |  |  |  |  |  | MYC | 6550600 | 4,122244 | up | 0,003771 |
|  |  |  |  |  |  | KCNS3 | 5080259 | 4,216919 | up | 0,003992 |
|  |  |  |  |  |  | CDC45L | 2320170 | 4,295058 | up | 0,009604 |
|  |  |  |  |  |  | AFMID | 2000497 | 4,32788 | up | 0,019825 |
|  |  |  |  |  |  | RPS26P10 | 620019 | 4,353341 | up | 0,000155 |
|  |  |  |  |  |  | SERPINB2 | 5810095 | 4,365831 | up | 0,015885 |
|  |  |  |  |  |  | FOS | 4280017 | 4,37364 | up | 0,048957 |
|  |  |  |  |  |  | KIF15 | 5360386 | 4,422399 | up | 0,000351 |
|  |  |  |  |  |  | PBK | 3420554 | 4,536635 | up | 0,0031 |
|  |  |  |  |  |  | SGCE | 5290348 | 4,652769 | up | 0,001783 |
|  |  |  |  |  |  | AURKB | 6770026 | 4,672781 | up | 0,002973 |
|  |  |  |  |  |  | TFF1 | 1230672 | 4,72737 | up | 0,013205 |
|  |  |  |  |  |  | LOC402644 | 6130259 | 4,917766 | up | 0,006966 |
|  |  |  |  |  |  | LOC641849 | 3850278 | 4,982842 | up | 0,002077 |
|  |  |  |  |  |  | LOC647436 | 2000367 | 5,004541 | up | 0,00526 |
|  |  |  |  |  |  | FOSB | 7160239 | 5,040818 | up | 0,024357 |
|  |  |  |  |  |  | NMU | 7050220 | 5,162199 | up | 0,016221 |
|  |  |  |  |  |  | COL17A1 | 2120270 | 5,238952 | up | 0,038224 |
|  |  |  |  |  |  | LOC645968 | 1440121 | 5,779814 | up | 0,00328 |
|  |  |  |  |  |  | LOC387934 | 830682 | 5,973427 | up | 0,001431 |
|  |  |  |  |  |  | NACAP1 | 3830136 | 6,068719 | up | 0,000537 |
|  |  |  |  |  |  | SERPINB2 | 6350209 | 6,1131 | up | 0,01769 |
|  |  |  |  |  |  | STATH | 20113 | 6,160229 | up | 0,009249 |
|  |  |  |  |  |  | KRT14 | 4900458 | 6,205206 | up | 0,017672 |
|  |  |  |  |  |  | LYZ | 4810162 | 6,312898 | up | 0,007106 |
|  |  |  |  |  |  | DMBT1 | 6220209 | 10,26692 | up | 0,009199 |
